# Linagliptin Ameliorates Hepatic Steatosis via Non-Canonical Mechanisms in Mice Treated with a Dual Inhibitor of Insulin Receptor and IGF-1 Receptor

**DOI:** 10.3390/ijms21217815

**Published:** 2020-10-22

**Authors:** Tomoko Okuyama, Jun Shirakawa, Kazuki Tajima, Yoko Ino, Heidrun Vethe, Yu Togashi, Mayu Kyohara, Ryota Inoue, Daisuke Miyashita, Jinghe Li, Nozomi Goto, Taiga Ichikawa, Shingo Yamasaki, Haruka Ohnuma, Rie Takayanagi, Yayoi Kimura, Hisashi Hirano, Yasuo Terauchi

**Affiliations:** 1 Department of Endocrinology and Metabolism, Graduate School of Medicine, Yokohama City University, Yokohama 236-0004, Japan; oku_tomo@yokohama-cu.ac.jp (T.O.); firsttrees2000@yahoo.co.jp (K.T.); yutogas@yokohama-cu.ac.jp (Y.T.); kyohara@yokohama-cu.ac.jp (M.K.); t176008a@yokohama-cu.ac.jp (R.I.); t176062f@yokohama-cu.ac.jp (D.M.); t176073c@yokohama-cu.ac.jp (J.L.); e143034d@yokohama-cu.ac.jp (N.G.); e133010d@yokohama-cu.ac.jp (T.I.); e123084d@yokohama-cu.ac.jp (S.Y.); e143014e@yokohama-cu.ac.jp (H.O.); e143050f@yokohama-cu.ac.jp (R.T.); terauchi@yokohama-cu.ac.jp (Y.T.); 2 Laboratory and Diabetes and Metabolic Disorders, Institute for Molecular and Cellular Regulation (IMCR), Gunma University, Maebashi 371-8510, Japan; 3 Advanced Medical Research Center, Yokohama City University, Yokohama 236-0004, Japan; yino@yokohama-cu.ac.jp (Y.I.); ykimura@yokohama-cu.ac.jp (Y.K.); 4 Department of Clinical Medicine, University of Bergen, P.O. Box 7803 Bergen, Norway; Heidrun.Vethe@uib.no; 5 Graduate School of Health Science, Gunma Paz University, Takasaki 370-0006, Japan; hirano@paz.ac.jp

**Keywords:** hepatic steatosis, insulin resistance, insulin signaling, diabetes, DPP-4 inhibitors, proteomics, phosphoproteomics

## Abstract

Abnormal hepatic insulin signaling is a cause or consequence of hepatic steatosis. DPP-4 inhibitors might be protective against fatty liver. We previously reported that the systemic inhibition of insulin receptor (IR) and IGF-1 receptor (IGF1R) by the administration of OSI-906 (linsitinib), a dual IR/IGF1R inhibitor, induced glucose intolerance, hepatic steatosis, and lipoatrophy in mice. In the present study, we investigated the effects of a DPP-4 inhibitor, linagliptin, on hepatic steatosis in OSI-906-treated mice. Unlike high-fat diet-induced hepatic steatosis, OSI-906-induced hepatic steatosis is not characterized by elevations in inflammatory responses or oxidative stress levels. Linagliptin improved OSI-906-induced hepatic steatosis via an insulin-signaling-independent pathway, without altering glucose levels, free fatty acid levels, gluconeogenic gene expressions in the liver, or visceral fat atrophy. Hepatic quantitative proteomic and phosphoproteomic analyses revealed that perilipin-2 (PLIN2), major urinary protein 20 (MUP20), cytochrome P450 2b10 (CYP2B10), and nicotinamide N-methyltransferase (NNMT) are possibly involved in the process of the amelioration of hepatic steatosis by linagliptin. Thus, linagliptin improved hepatic steatosis induced by IR and IGF1R inhibition via a previously unknown mechanism that did not involve gluconeogenesis, lipogenesis, or inflammation, suggesting the non-canonical actions of DPP-4 inhibitors in the treatment of hepatic steatosis under insulin-resistant conditions.

## 1. Introduction

The prevalence of patients with non-alcoholic fatty liver disease (NAFLD) and non-alcoholic steatohepatitis (NASH), which are associated with diabetes and metabolic syndrome, have increased considerably [1,2]. Hepatic insulin action through growth hormone receptors-mediated signaling is involved in the development of fatty liver [3]. Fat accumulation in the liver has also been found to cause hepatic insulin resistance [4].

Insulin receptor (IR) and IGF-1 receptor (IGF1R) play roles in systemic metabolic actions, cell proliferation and migration, as well as cancer growth and metastasis [5,6]. Previously, a number of anti-IGF-1 receptor drugs, including monoclonal antibodies and tyrosine kinase inhibitors, have been developed as anti-tumor drugs [7,8]. OSI-906 (linsitinib) is an orally bioavailable dual IR/IGF1R tyrosine kinase inhibitor [9]. OSI-906 specifically inhibits the autophosphorylation of IR/IGF1R and their downstream pathways, resulting in the induction of insulin resistance. We previously reported that the oral administration of OSI-906 for 7 days induced glucose intolerance, liver steatosis, and lipoatrophy in mice [10,11]. In this model, insulin signaling in the liver was completely abolished [12].

Linagliptin, a selective dipeptidyl peptidase-4 (DPP-4) inhibitor, is mainly excreted in feces, though most DPP-4 inhibitors are cleared by the kidneys [13]. The enzymatic activity of incretin peptide, glucagonlike peptide 1 (GLP-1), and glucose-dependent insulinotropic polypeptide (GIP) are diminished by DPP-4 [14]. The functions of incretins are thought to potentiate glucose-dependent insulin secretion from pancreatic β-cells, inhibiting glucagon secretion; this in turn reduces hepatic gluconeogenesis. The inhibition of DPP-4 extends the half-life of endogenous active forms of GIP and GLP-1, and lowers hyperglycemia in patients with type 2 diabetes. In addition to their glucose-lowering effects, DPP-4 inhibitors reportedly have multiple pleiotropic effects that are independent of its pancreatic effects. Reportedly, DPP-4 inhibitors might ameliorate NAFLD in patients with type 2 diabetes [15,16]. DPP-4 inhibitors also reduced hepatic fat accumulation in experimental models of NAFLD [17,18]. However, the mechanisms responsible for the protective effects of DPP-4 inhibition on fatty liver are obscure. 

In this study, we administered the DPP-4 inhibitor linagliptin to OSI-906-injected mice to investigate whether linagliptin ameliorates fatty liver under the conditions of IR and IGF1R inhibition.

## 2. Results

### 2.1. Linagliptin Reduced Plasma Triglyceride Levels without Altering Blood Glucose and Serum Insulin Levels in OSI-906-Treated Mice

**Figure 1:**
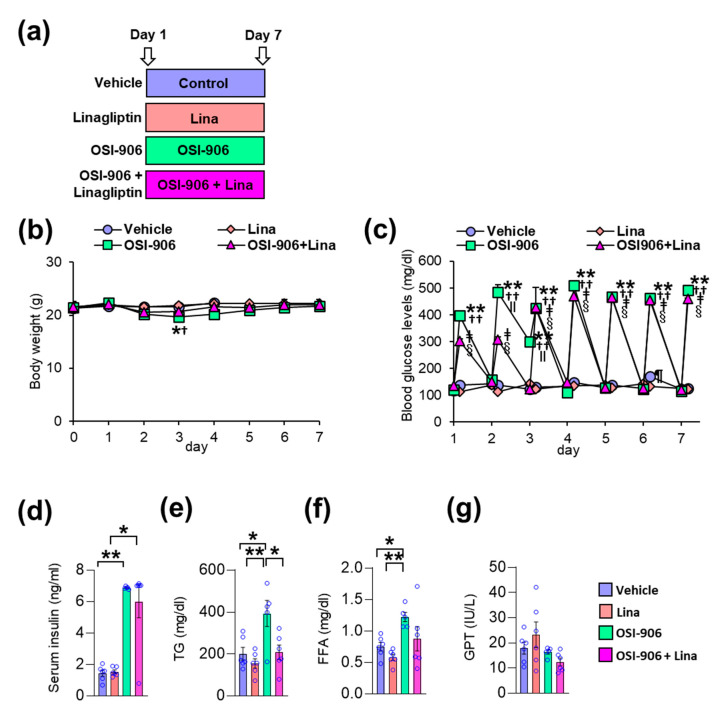
Linagliptin (Lina) improved OSI-906-induced hypertriglyceridemia in mice after 7 days of OSI-906 administration. (**a**) Experimental protocol (*n* = 5–6). (**b**) Body weight during the experiment. Data represent the mean ± SEM. * *p* < 0.05, OSI-906 vs. vehicle; ^†^ *p* < 0.05, OSI-906 vs. Lina by repeated measures ANOVA followed by Bonferroni multiple comparison test (*n* = 5–6 per group). (c) Blood glucose levels determined just before and 4 h after the administration of OSI-906 or the vehicle during the experiment. ** *p* < 0.01, OSI-906 vs. vehicle; ^††^ *p* < 0.01, OSI-906 vs. Lina, ^ǂ^ *p* < 0.01, OSI-906 + Lina vs. vehicle; ^§^ *p* < 0.01, OSI-906 + Lina vs. Lina; ^ǁ^ *p* < 0.01, OSI-906 + Lina vs. OSI-906; ^¶^ *p* < 0.01, Lina vs. vehicle by repeated measures of ANOVA followed by Bonferroni multiple comparison test (*n* = 5–6 per group). (d) Serum insulin, (e) serum triglyceride (TG), (f) serum free fatty acid (FFA), and (g) serum glutamic pyruvic transaminase (GPT) levels on day 7. Data represent the mean ± SEM. * *p* < 0.05, ** *p* < 0.01 (*n* = 5–6 per group) by ANOVA with an additional Tukey–Kramer post-hoc test.

In this study, wild-type C57BL/6J mice were orally injected with OSI-906 at a dosage of 45 mg/day, thereby blunting IR- and IGF1R-mediated signaling in the liver and white adipose tissue [10,12]. Linagliptin was administered by oral gavage at a dosage of 3 mg/kg/day, thereby significantly inhibiting DPP-4 activity and significantly increasing the GLP-1 level [17]. The mice were treated with the vehicle, linagliptin (Lina), OSI-906, or OSI-906 in combination with linagliptin (OSI-906 + Lina) for 7 days (Figure 1a). The oral administration of OSI-906 transiently decreased the body weight observed on day 3 (Figure 1b). Linagliptin prevented the OSI-906-induced reductions in body weight observed on day 3 (Figure 1b). Blood glucose levels were significantly elevated at 4 h after OSI-906 administration, and linagliptin partially reduced the blood glucose levels observed on day 2 (Figure 1c). However, hyperglycemia elicited by OSI-906 administration did not improve with linagliptin treatment after day 3 (Figure 1c).

On day 7, the serum insulin levels became significantly higher after OSI-906 administration, consistent with the inhibition of IR/IGF1R. Treatment with linagliptin did not influence the hyperinsulinemia observed in mice treated with OSI-906 (Figure 1d). On the other hand, linagliptin canceled the OSI-906-induced elevation in plasma triglyceride levels, although no significant differences in plasma free fatty acid (FFA) levels were observed between the OSI-906 and OSI-906 + Lina groups (Figure 1e,f). The serum glutamic pyruvic transaminase (GPT) levels were not altered by the administration of OSI-906 or by treatment with linagliptin (Figure 1g).

### 2.2. Linagliptin Improved OSI-906-Induced Hepatic Steatosis

**Figure 2:**
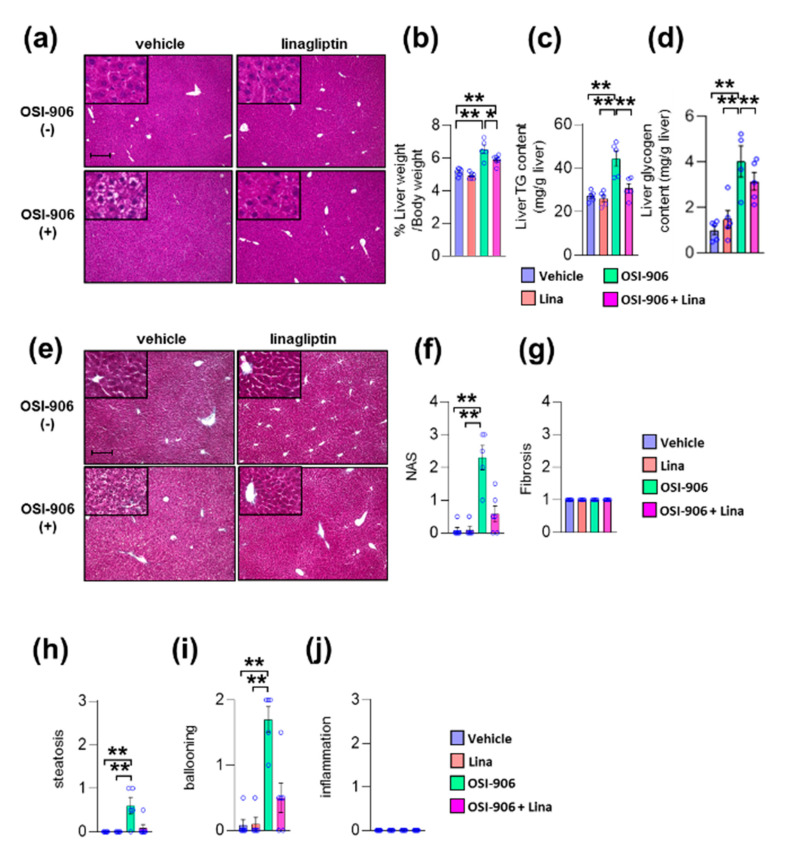
Linagliptin improved hepatic steatosis evoked by OSI-906. (**a**) Hematoxylin and eosin-stained sections of liver on day 7. Scale bar = 200 μm. (**b**) Ratio of liver weight to body weight on day 7. (**c**,**d**) Triglyceride (TG) and glycogen content in the liver on day 7. Data represent the mean ± SEM. * *p* < 0.05, ** *p* < 0.01 (*n* = 5 per group) by ANOVA with an additional Tukey–Kramer post-hoc test. (**e**) Masson–Goldner-stained section of liver on day 7. Scale bar = 200 μm. (**f**) Non-alcoholic fatty liver disease (NAFLD) activity score (NAS), (**g**) fibrosis staging, and (**h**) the degree of steatosis, (**i**) hepatocyte ballooning, (**j**) lobular inflammation of liver sections according to the NAFLD activity score (NAS) score. Data represent the mean ± SEM. *p < 0.05, ** *p* < 0.01 (*n* = 4–6 per group) by Kruskal–Wallis test.

We previously reported that OSI-906 administration induced lipoatrophy and hepatic steatosis after 7 days of administration in wild-type mice [10]. We also reported that DPP-4 inhibition prevented diet-induced adipose tissue inflammation and hepatic steatosis in diabetic mice [19]. Next, we investigated the impact of DPP-4 inhibition on lipoatrophy or hepatic steatosis in wild-type mice treated with OSI-906 for 7 days. The atrophic changes in visceral fat elicited by OSI-906 were not affected by the treatment with linagliptin (Supplementary Figure S1a,b). In contrast, DPP-4 inhibition with linagliptin improved OSI-906-induced hepatic steatosis (Figure 2a). Thus, we further assessed the effects of linagliptin on the liver in OSI-906-treated mice. The administration of OSI-906 significantly increased the liver weight, and this increase in liver weight was significantly lower in the OSI-906 + Lina group than in the OSI-906 group (Figure 2b). The hepatic triglyceride content and the hepatic glycogen content were significantly increased in the OSI-906 group, whereas these parameters were reversed by the treatment with linagliptin (Figure 2c,d). In addition, the NAFLD activity score (NAS) [20], a score for the severity of steatosis, inflammation, and hepatocyte ballooning, was significantly increased by the administration of OSI-906 and tended to be restored by the treatment with linagliptin, consistent with the protective effect of linagliptin against OSI-906-induced hepatic steatosis (Figure 2e–j). Fibrosis was also determined using fibrosis staging [21]. Notably, OSI-906 did not induce inflammation and fibrosis in the liver in spite of the development of steatosis and ballooning (Figure 2e–j). 

**Figure 3:**
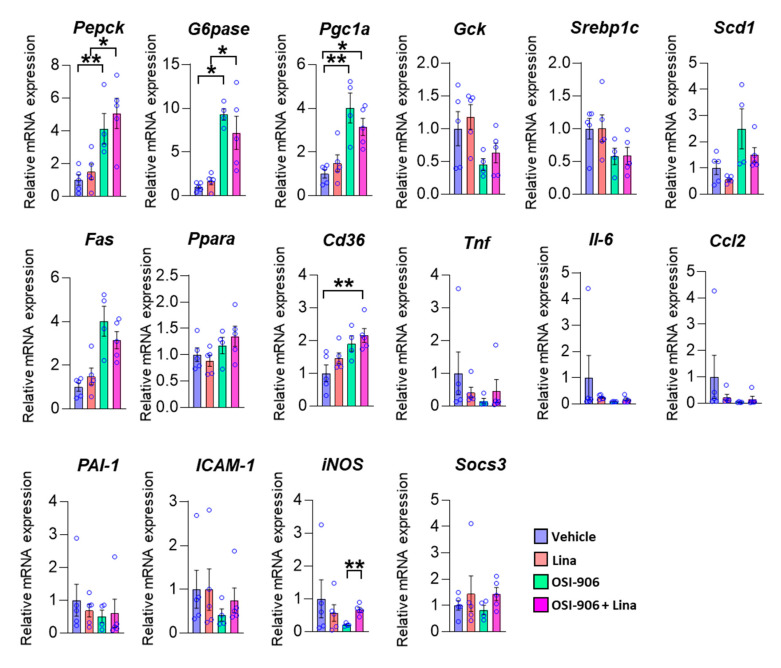
Hepatic gene expressions of the indicated molecules on day 7. Data represent the mean ± SEM. * *p* < 0.05, ** *p* < 0.01 (*n* = 4–5 per group) by ANOVA with an additional Tukey–Kramer post-hoc test.

We next examined the hepatic gene expression involved in hepatic metabolism on day 7 (Figure 3). The expressions of gluconeogenic genes, such as *G6pase* and *Pepck*, and a potent activator of gluconeogenesis, *Pgc-1α*, were increased in the liver by the OSI-906 administration, consistent with the impairment of hepatic insulin action caused by the blocking of IR and IGF1R. The treatment with linagliptin did not change these expressions. The expressions of *Srebp1c*, *Fas*, and *Scd1*, which are involved in de novo lipogenesis, were not altered in the presence of linagliptin in OSI-906-treated mice. There were no significant differences in gene expressions of *Gck* and *Ppara*, which are involved in insulin action. In contrast, the expression of *Cd36* was increased in the OSI-906 + Lina group compared with the vehicle control, although there were no significant differences between the OSI-906 group and the OSI-906 + Lina group. We also investigated the expressions of genes related to inflammation and oxidative stress in the liver because these processes are closely related to the development of hepatic steatosis or NAFLD (Figure 3). Interestingly, the expressions of inflammatory genes, such as *Tnf-a*, *Il-6*, *Ccl2*, *ICAM-1*, and *iNos*, tended to be reduced by the administration with OSI-906 for 7 days. These results were consistent with the lack of inflammatory changes in the liver treated with OSI-906 in Figure 2. Linagliptin showed the tendency to restore the reductions in the expression of *Tnf-a*, *Il-6*, and *Ccl2* in the liver. The administration of OSI-906 and treatment with linagliptin did not change *PAI-1* and *Socs3* expressions.

### 2.3. Hepatic Proteomic and Phosphoproteomic Analyses Revealed Insulin- and Glucose-Independent Effects of Linagliptin in OSI-906-Induced Hepatic Steatosis

**Figure 4:**
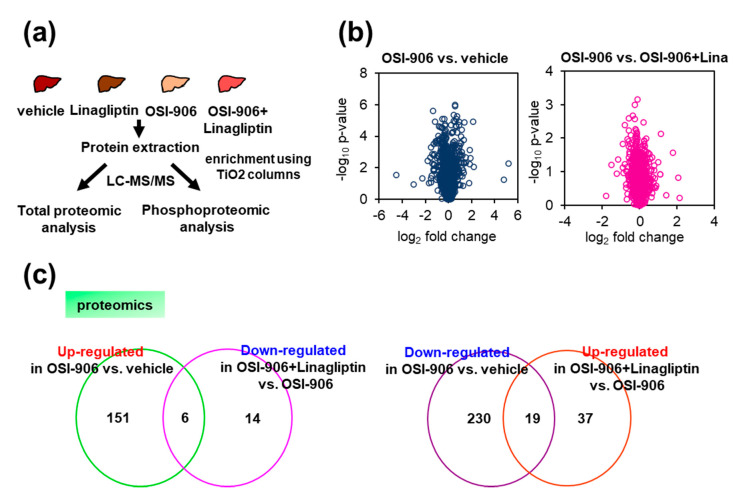
Proteomic and phosphoproteomic analysis of the liver from OSI-906- and linagliptin-treated mice. (**a**) Protocol for proteomic and phosphoproteomic analyses using liver samples (*n* = 5 per group). (**b**) Volcano plot of hepatic proteomic data. Molecules identified in comparisons of vehicle vs. OSI-906 (left) and OSI-906 vs. OSI-906 + linagliptin (right) are shown (*n* = 5 per group). (**c**) Venn diagram of differentially expressed molecules in OSI-906 vs. vehicle and OSI-906 + Lina vs. OSI-906 in proteomic analyses of liver samples. The numbers of proteins that were significantly upregulated or downregulated (ANOVA *p*-value < 0.05, fold change > 1.2) compared with the respective controls are shown (*n* = 5 per group). The lists of overlapping proteins are shown in Supplementary Table S1.

We also performed hepatic quantitative proteomic and phosphoproteomic analyses of the livers from OSI-906- or linagliptin-treated mice (Figure 4a). We identified a total of 1884 proteins, and differentially expressed proteins were analyzed using Progenesis QI software to compare expressions in the OSI-906 vs. vehicle groups and the OSI-906 + Lina vs. OSI-906 groups. Scatter plots (volcano plots) that display the *p*-value versus the fold change for OSI-906 relative to the vehicle or OSI-906 relative to OSI-906 + Lina highlighted differently expressed proteins by OSI-906 or linagliptin in the liver (Figure 4b). 

We focused on proteins with expressions that were significantly altered by the administration of OSI-906 and for which the alterations were reversed by the treatment with linagliptin (Figure 4c and Supplementary Table S1) to address the amelioration of hepatic steatosis by linagliptin in the OSI-906-treated model. Among the proteins that were significantly upregulated or downregulated in response to the administration of OSI-906 (Supplementary Tables S1–S3), the abundances of perilipin-2 (PLIN2) and cytochrome P450 2b10 (CYP2B10) were reduced by OSI-906 and were restored by treatment with linagliptin. In contrast, the abundance of major urinary protein 20 (MUP20) was increased by OSI-906 and was also restored by treatment with linagliptin. Those results indicate that these molecules might contribute to the process of the linagliptin-induced amelioration of hepatic steatosis.

A previous study reported that the expressions of cytochrome P450 enzymes were downregulated and those of major urinary proteins (MUPs) were upregulated by treatment with resveratrol, a sirtuin activator, in the liver [22]. We also found that the protein expression of nicotinamide N-methyltransferase (NNMT) was significantly upregulated in OSI-906-treated liver. NNMT metabolizes the nicotinamide adenine dinucleotide (NAD^+^) precursor nicotinamide (NAM) and the methyl donor S-adenosylmethionine (SAM) [23]. Thus, NNMT activity is involved in the NAD^+^-dependent enzymes and the SAM-dependent methyltransferases.

**Figure 5:**
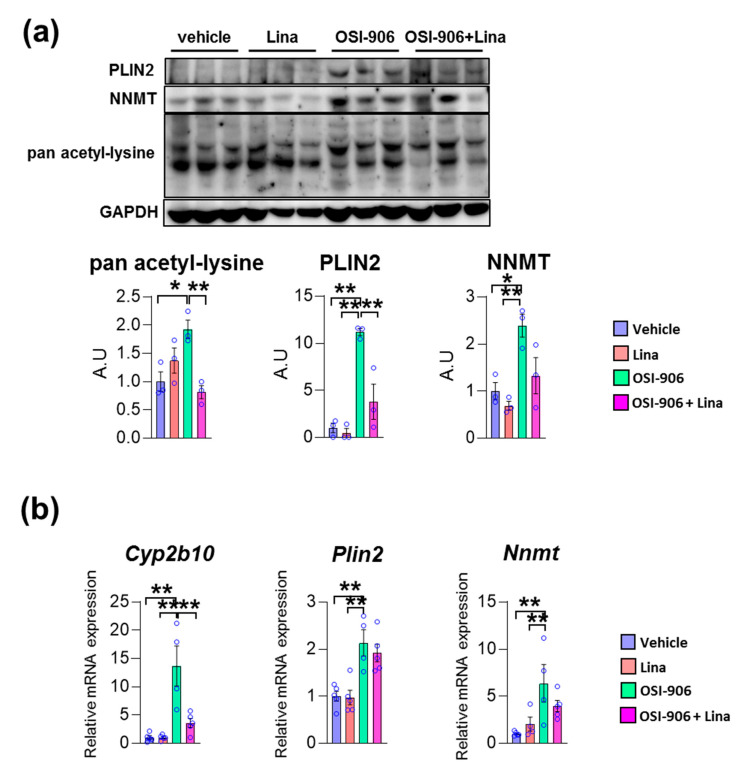
Linagliptin reversed the increased expression of acetylated lysine, perilipin-2, and nicotinamide N-methyltransferase (NNMT) in OSI-906-treated liver. (**a**) Immunoblotting for pan acetyl-lysine, perilipin-2, and NNMT in the liver (*n* = 3 per group). Densitometry was plotted in lower graphs. (**b**) Hepatic gene expressions of *Cyp2b10, Plin2,* and *Nnmt* on day 7. Data represent the mean ± SEM. * *p* < 0.05, ** *p* < 0.01 (*n* = 4–5 per group) by ANOVA with an additional Tukey–Kramer post-hoc test.

We validated these findings using immunoblotting or gene expression analyses. The protein expressions of PLIN2 was increased in OSI-906-treated liver, and its increase was reversed by linagliptin (Figure 5a). Similarly, OSI-906 increased the NNMT protein level and the treatment with linagliptin partially reversed its elevation. In addition, the increased pan acetyl-lysine levels in OSI-906-treated liver were also reversed by linagliptin. Since sirtuins are NAD^+^-dependent lysine deacetylases [24], these results imply an enhancement of sirtuin activity by linagliptin in this OSI-906-induced hepatic steatosis model (Figure 5a). The hepatic mRNA expression of *Plin2* was increased by the administration of OSI-906, whereas linagliptin did not reduce its expression (Figure 5b). In addition, hepatic mRNA expression of *Nnmt* was also induced by OSI-906. Linagliptin showed the tendency to attenuate increase in *Nnmt* expression in the liver, though it did not reach to the statistical significance. The hepatic gene expression of *Cyp2b10* was also induced by OSI-906 and was deceased by treatment with linagliptin (Figure 5b).

To address whether sirtuins are involved in the effect of linagliptin on OSI-906-induced hepatic steatosis, we investigated protein expression of sirtuins and its deacetylase activity in the liver. SIRT1 and SIRT2 expression were significantly enhanced by linagliptin in the liver (Supplementary Figure S2a). However, the administration of OSI-906 did not reduced those levels compared with the vehicle group, and the impact of linagliptin on SIRT1 and SIRT2 expressions was abolished by the administration of OSI-906. Both OSI-906 and linagliptin did not influenced on phosphorylation of AMP-activated protein kinase (AMPK) in the liver (Supplementary Figure S2a). In hepatocyte AML-12 cells, SIRT1 and SIRT2 expressions were also not diminished by the administration of OSI-906 (Supplementary Figure S2b). Although treatment with linagliptin tended to increase SIRT1 and SIRT2 protein expression in AML-12 cells, the ratio of phosphorylation in AMPK was not increased after treatment with linagliptin (Supplementary Figure S2b). In contrast, treatment with liraglutide restored AMPK phosphorylation in OSI-906-treated AML-12 cells. Next, we examined the level of acetylated lysine in PGC-1α and FoxO1 in the liver to address sirtuin deacetylase activity. OSI-906 did not increase acetylated lysine in PGC-1α and FoxO1, and linagliptin did not influence on those expressions, implying that sirtuin deacetylase activity may not directly be involved in the effect of linagliptin or OSI-906 in this model. Thus, those results indicate that linagliptin ameliorates OSI-906-induced hepatic steatosis possibly through the NNMT-dependent and sirtuin-independent pathway.

**Figure 6:**
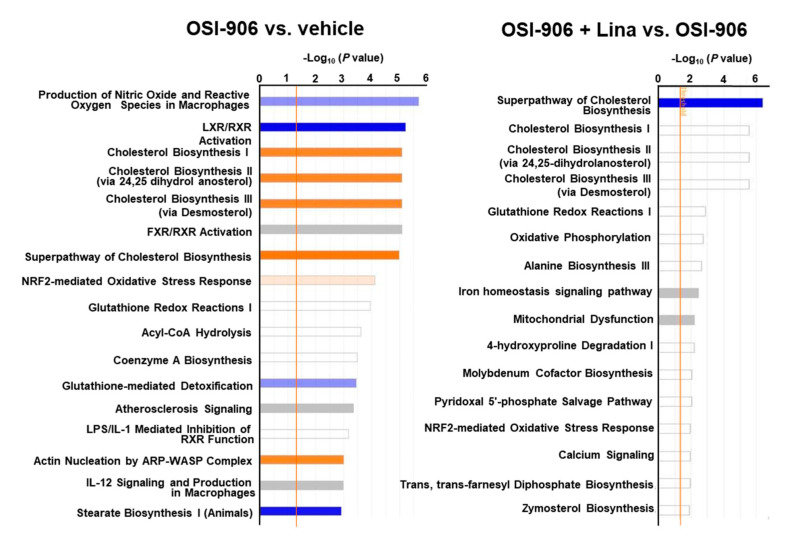
Canonical pathway analysis of proteomics in the livers of OSI-906- and linagliptin-treated mice. A canonical pathway analysis of the hepatic proteomic data was performed using molecules that were differentially expressed (ANOVA *p* < 0.05) in comparisons of OSI-906 vs. vehicle and OSI-906 + Lina vs. OSI-906. The color of the bar indicates the z-score (orange, positive z-score; blue, negative z-score). The orange line depicts the statistical significance threshold (*p* = 0.05).

A canonical pathway analysis of differentially expressed proteins in the OSI-906 vs. vehicle groups and the OSI-906 + Lina vs. OSI-906 groups revealed that the administration of OSI-906 activated the cholesterol biosynthesis pathway and inhibited the LXR/RXR pathway, whereas linagliptin inhibited the cholesterol biosynthesis pathway (Figure 6).

In a phosphoproteomic analysis of liver samples from the four groups, more than 3700 sites of phosphorylation were identified. The top increased or decreased phosphopeptides when the OSI-906 vs. vehicle groups or the OSI-906 + Lina vs. OSI-906 groups were compared are shown in Supplementary Tables S4–S7. 

**Figure 7:**
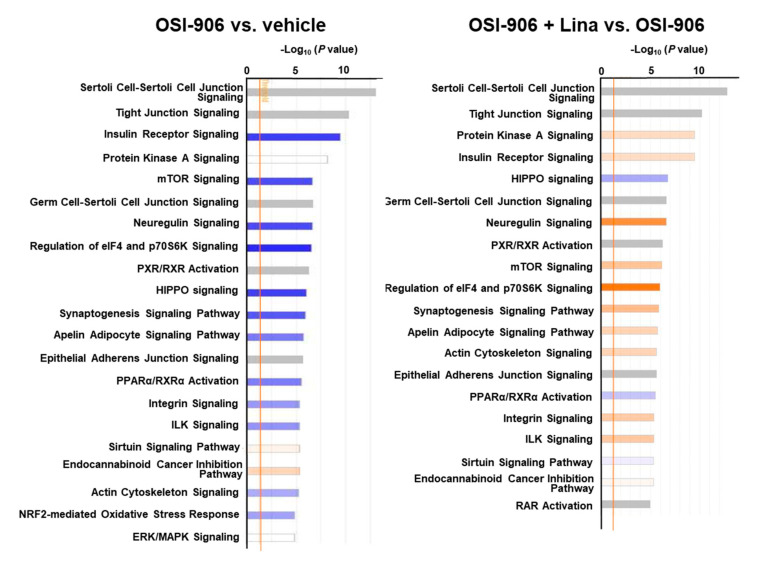
Canonical pathway analysis of phosphoproteomics in the livers of OSI-906- and linagliptin-treated mice. A canonical pathway analysis of the hepatic phosphoproteomic data was performed using molecules that were differentially expressed (ANOVA *p* < 0.05) in comparisons of OSI-906 vs. vehicle or OSI-906 + Lina vs. OSI-906. The color of the bar indicates the z-score (orange, positive z-score; blue, negative z-score). The orange line depicts the statistical significance threshold (*p* = 0.05).

A canonical pathway analysis of the phosphoproteomics data identified the inhibition of insulin receptor signaling and mTOR signaling in OSI-906-treated liver, consistent with the dual inhibition of insulin/IGF-1 receptors by OSI-906 (Figure 7). Treatment with linagliptin did not seem to affect insulin signaling. An upstream regulator analysis indicated that PPARα and XBP1 were the top 2 predicted transcription activators involved in the process of hepatic steatosis induced by OSI-906, whereas pathways related to SREBF2 and lysophosphatidylcholine were identified in the linagliptin-treated group (Supplementary Table S8).

### 2.4. Linagliptin Exerts Direct and Indirect Effects on Hepatocytes during the Improvement of Hepatic Steatosis

**Figure 8:**
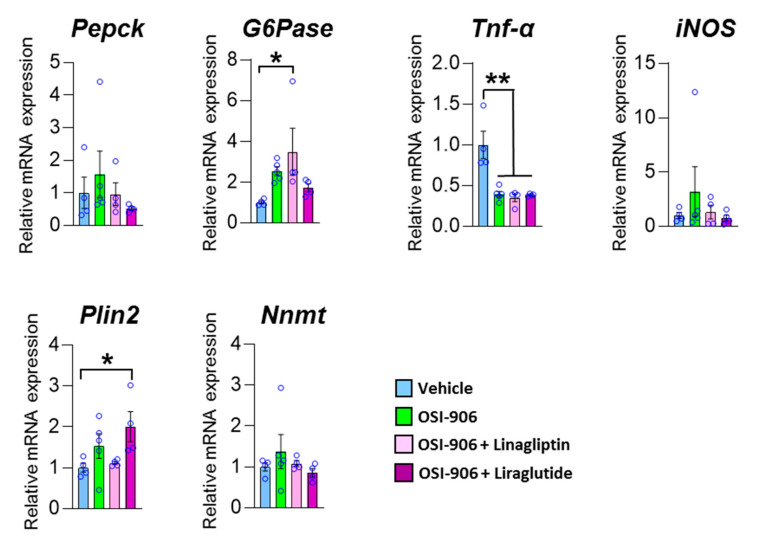
Impact of linagliptin or liraglutide on gene expressions in OSI-906-treated AML-12 cells. The gene expressions of the indicated genes in AML-12 cells treated with 10 nM linagliptin or 100 nM liraglutide in the presence of 200 nM OSI-906 are shown. Cells were serum-starved overnight and then incubated with OSI-906 for 4 h before the treatment with linagliptin or liraglutide. Then, the cells were incubated for 24 h with the indicated drugs. Data represent the mean ± SEM. * *p* < 0.05, ** *p* < 0.01 (*n* = 4–5 per group) by ANOVA with an additional Tukey–Kramer post-hoc test.

To elucidate whether the effect of linagliptin on hepatic steatosis was mediated through the direct action of linagliptin, GLP-1 receptor signaling in hepatocytes, or an indirect pathway through components such as humoral factors, we treated AML-12 cells with the DPP-4 inhibitor linagliptin or the GLP-1 receptor agonist liraglutide in the presence of OSI-906 (Figure 8). In AML-12 hepatocyte cells, linagliptin did not reverse the effects of OSI-906 on gluconeogenic gene expressions, consistent with the *in vivo* results. In contrast, the decrease in *Tnf* expression in OSI-906-treated cells was not reversed by incubation with linagliptin or liraglutide, unlike the *in vivo* results, implying an indirect effect of linagliptin and DPP-4 inhibition on hepatocytes. In contrast, liraglutide tended to reverse the OSI-906-induced increases in gluconeogenic gene expressions. The expression of *Plin2* tended to be increased by OSI-906 and to be diminished by the treatment with linagliptin. In contrast, the treatment with liraglutide increased the expression of *Plin2* in AML-12 cells, implying that linagliptin effects hepatocyte via a GLP-1 receptor signaling-independent manner. These results indicate that linagliptin influences hepatocytes through multiple mechanisms that are independent of GLP-1 receptor signaling.

## 3. Discussion

In this study, we reported that the DPP-4 inhibitor linagliptin ameliorated hepatic steatosis elicited by the dual IR/IGF1R inhibitor OSI-906. We previously reported that a DPP-4 inhibitor, des-fluoro-sitagliptin, prevented diet-induced adipose tissue inflammation and hepatic steatosis [25]. In another study, linagliptin reportedly improved insulin sensitivity and hepatic steatosis in diet-induced obese (DIO) mice [17]. DPP-4 inhibition with linagliptin has been shown to decrease the expressions of SREBP1c, SCD-1, and FAS, all of which are known to be related to *de novo* lipogenesis, and increased the expression of PPARα in the livers of DIO mice [17]. On the other hand, the expressions of genes involved in hepatic fatty acid oxidation were not affected by treatment with linagliptin in DIO or NASH mouse models [17,18]. However, the mechanism by which DPP-4 inhibitors improve fatty liver in the presence of diet-induced obesity or diabetes remains unclear.

IR- and IGF-1R-mediated signaling play a crucial role in hepatic insulin action, which regulates glucose and fatty acid metabolism. OSI-906 administration induces the acute inhibition of IR/IGF1R signaling and evokes hepatic steatosis [10]. In our mouse model, the dual inhibition of IR and IGF-1R with OSI-906 resulted in impaired insulin signaling in the liver. The liver weight and liver TG contents were significantly increased by the administration of OSI-906 for 7 days. Linagliptin significantly reversed the OSI-906-induced increase in liver weight and TG content. The administration of OSI-906 increased hepatic glycogen content despite impaired hepatic insulin signaling. Multiple insulin-regulated enzymes, such as glucokinase (GCK) and glycogen synthase, participate in hepatic glycogen synthesis. Importantly, the administration of OSI-906 reduced *Gck* expression in the liver. The expression of glycogen synthase in the liver was not altered by a week of OSI-906 administration to the wild-type mice in our previous study [10]. These results indicated that the accumulation of glycogen in the liver was independent of glucokinase, glycogen synthase, or insulin signaling in OSI-906 treated mice. The increase in hepatic glycogen content in those mice was partially reversed by linagliptin, potentially through a transient reduction in blood glucose levels, a slight recovery of hepatic insulin signaling (shown in Figure 7) by linagliptin. The OSI-906 group exhibited increased gluconeogenic gene expression, compared with the vehicle group, and treatment with linagliptin did not affect the expressions of these genes, consistent with an impairment in hepatic insulin action. The expressions of *Srebp1c*, *Fas*, and *PPARα*, which were also regulated by insulin signaling, showed no significant alteration by treatment with linagliptin in OSI-906-treated mice. Hence, the effect of linagliptin on hepatic steatosis seemed to be independent of the IR/IGF1R signaling pathway. Our data indicated that although insulin signaling is involved in the development of hepatic steatosis, an alternative pathway that improves liver steatosis without altering insulin signals likely exists in the OSI-906-treated model. The OSI-906 + Lina group had lower glucose levels at 4 h after OSI-906 administration on day 2, compared with the levels in the OSI-906 group; however, these effects were abolished after day 3. Hyperinsulinemia evoked by OSI-906 was also observed in the OSI-906 + Lina group. Therefore, the glucose-lowering effects of linagliptin did not contribute to the improvement in OSI-906-induced hepatic steatosis. These results imply that linagliptin improved OSI-906-induced hepatic steatosis without glucose lowering effects or reduction in hyperinsulinemia.

In patients with metabolic syndrome, fatty liver occurs because of an increased uptake of fatty acids accompanied by an impairment in insulin action in adipose tissue. In this study, linagliptin did not reduce the plasma free fatty acid levels and hepatic expression of *Cd36*, which is a free fatty acid transporter, in OSI-906-treated mice. Therefore, linagliptin seemed to ameliorate OSI-906-induced hepatic steatosis independently of the fatty acid flux to the liver, consistent with the regulation of fatty acid metabolism via insulin. Thus, linagliptin improved OSI-906-induced hepatic steatosis via a pathway that was independent of insulin signaling, glucose levels, or free fatty acid metabolism.

Liver-specific IR knockout (LIRKO) mice showed severe insulin resistance and glucose intolerance with increased hepatic glucose production, impaired glucose utilization in the liver, and abnormal mitochondrial function because of the failure of insulin activity [3,26,27]. The loss of FoxO1 increases lipogenesis and decreases fatty acid oxidation in hepatocytes. Thus, liver FoxO1-null mice developed hepatic steatosis, accompanied by the upregulation of lipogenic genes. This regulation of lipogenesis by FoxO1 requires intact hepatic insulin signaling. The systemic inhibition of IR with S961 blunted the effects of insulin on hepatic glucose production in liver-specific IR/FoxO1 double knockout LIRFKO mice [28]. Thus, systemic insulin signaling might be required to regulate hepatic glucose production and de novo lipogenesis. On the other hand, lipodystrophy in adipose tissue-specific IR knockout (F-IRKO) mice causes progressive NAFLD [29]. Treatment with OSI-906 also induced lipodystrophy and hepatic steatosis, similar to the F-IRKO mouse model. These findings indicate that not only hepatic insulin signaling, but also insulin signaling in adipose tissue, plays a crucial role in the development in hepatic steatosis. However, in this study, linagliptin did not reverse the lipodystrophy induced by OSI-906 administration, implying that linagliptin exerts its effect on OSI-906-induced hepatic steatosis without altering either hepatic insulin signaling or insulin signaling in adipose tissue.

Chronic low-grade inflammation is known to contribute to the development of steatosis [30]. Interestingly, the inhibition of IR and IGF1R by OSI-906 showed a tendency to decrease inflammatory gene expressions. These results indicate that OSI-906-induced hepatic steatosis is not provoked by the inflammatory response, unlike high-fat diet-induced hepatic steatosis. We previously reported that DPP-4 inhibition prevented diet-induced hepatic steatosis and adipose tissue inflammation in a diabetic mouse model [31]. Linagliptin reportedly reduced advanced glycation end product (AGE)-related oxidative stress in the kidneys of a type 1 diabetes mouse model [32], whereas no significant differences in the expressions of genes related to oxidative stress were observed in the current study. Thus, in this OSI-906-induced hepatic steatosis model, the identification of a new molecular mechanism for hepatic steatosis via insulin resistance, other than inflammation or oxidative stress, might be useful for understanding the development of hepatic steatosis.

Proteomic and phosphoproteomic analyses revealed some possible mechanisms underlying OSI-906-induced progression in hepatic steatosis and its amelioration by treatment with linagliptin. We focused on molecules such as PLIN2 and NNMT, which were suggested by a proteomic analysis to be responsible for the effect of linagliptin on hepatic steatosis. PLIN2, a lipid droplet-coating protein, is related to lipid accumulation in the liver and promotes hepatic steatosis [33,34,35]. PPAR γ is also known to be required for the induction of PLIN2. In a quantitative proteomic analysis, OSI-906 administration significantly upregulated PLIN2, whereas treatment with linagliptin almost canceled the OSI-906-induced elevation in PLIN2. These results imply that the regulation of PLIN2 in the liver is associated with the development of hepatic steatosis induced by OSI-906 and its recovery as a result of DPP-4 inhibition using linagliptin. Although mRNA expression of *Plin2* was not statistically different between the OSI-906 group and the OSI-906 + Lina group, it is possible that the protein levels of PLIN2 might be regulated via translation, protein stability, or protein degradation, in addition to gene transcription. Similarly, *Cyp2b10* was upregulated in OSI-906-treated liver, and treatment with linagliptin reversed this induction. Regarding MUP20, its expression was reduced by OSI-906 and restored by linagliptin. The regulation of Cyp2b10 and Mup20 were consistent with the context reported for high-fat-diet-induced steatosis and its recovery by treatment with resveratrol [22]. Cyp2b10 is related to lipid metabolism in the liver, and one of the regulators of Cyp2b10 is a constitutive androgen receptor (CAR) [36]. Major urinary proteins, which are secreted proteins, belong to the lipocalin family and are predominantly produced by the liver and excreted in urine [37]. MUPs play a role as carriers of pheromones [37] and act as a pheromone itself. A previous report showed that MUP1 increases energy expenditure and improves glucose tolerance [38].

There were no previously known direct interactions among the molecules, which we focused on in this study, in the STRING database (https://string-db.org/). A previous report showed that systemic ablation of *Sirt1* in mice increased the expression of *Plin2* in adipose tissue [39]. Thus, the regulation of sirtuin activity might regulate *Plin2* expression. Several reports showed the possible association between DPP-4 inhibitors and sirtuin activity [40,41]. In this study, our findings indicate the impact of linagliptin on NNMT expression. In fact, we found increased SIRT1 and SIRT2 expression in linagliptin-treated liver. However, OSI-906 seemed to not attenuate sirtuin expression or its activity in this study. Moreover, we did not find the alteration in sirtuin activity after treatment with linagliptin. Therefore, linagliptin might regulate the expression of NNMT via the sirtuin-independent pathway that requires further investigation.

There are several signal transduction pathways that have been proposed to improve hepatic steatosis as a result of incretin-based therapy, such as cAMP-PKA signaling, PI3K-PDK1-Akt/PKB signaling, and AMPK signaling [15]. Whether incretin exerts direct effects or facilitates indirect pathways affecting hepatocyte metabolism remains controversial. Moreover, a recent report showed the importance of hepatocyte-derived DPP-4 and obesity on adipose inflammation and insulin resistance [42].

To address the conflicts in the actions of DPP-4 inhibitors and the GLP-1 receptor agonist on the liver, we examined the effect of linagliptin and the GLP-1 analog liraglutide on hepatocyte AML-12 cells. Linagliptin did not inhibit gluconeogenic gene expressions in OSI-906-treated AML-12 cells, consistent with an insulin signaling-independent action of linagliptin on hepatic steatosis. On the other hand, liraglutide showed a tendency to reduce the expressions of gluconeogenic genes in OSI-906-treated cells. These results suggest that the DPP-4 inhibitor and the GLP-1 analog had different effects on insulin signaling in hepatocytes. In addition, the influence of linagliptin on *Tnf* expression differed between *in vivo* and *in vitro* studies, implying an indirect action of linagliptin on hepatocytes that does not involve the regulation of DPP-4 enzymatic activity. Previously, we reported the protective effects of liraglutide on hepatic steatosis in β-cell-specific glucokinase-deficient mice with severe defects of insulin secretion [43]. Because OSI-906 also blunts insulin signaling as well as insulin deficiency, liraglutide may have a protective effect on steatosis in the OSI-906-treated model.

**Figure 9:**
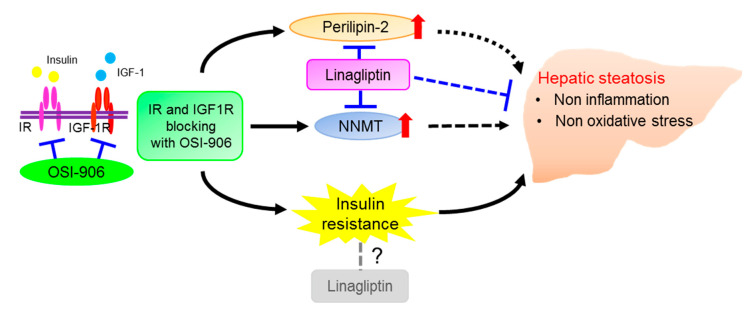
Schematic regulatory pathway of linagliptin in OSI-906-induced hepatic steatosis. Linagliptin improved hepatic steatosis induced by acute IR/IGF1R signaling inhibition with OSI-906 through an insulin signaling-independent pathway. Pathways involving perilipin-2 and NNMT have been proposed as possible mechanisms for the amelioration of OSI-906-induced hepatic steatosis by linagliptin.

Taken together, these results show an effect of DPP-4 inhibition on hepatic steatosis that is induced by the acute inhibition of IR/IGF1R signaling through an insulin signaling-independent pathway (Figure 9). Our findings support the non-canonical pleiotropic effects of DPP-4 inhibitors without a glucose-lowering effect and suggest the potential of DPP-4 inhibitors as a new treatment for fatty liver disease. Further investigation of the underlying mechanism is required.

## 4. Materials and Methods

### 4.1. Animals and Animal Care

C57BL/6J male mice (CLEA Japan, Tokyo, Japan) aged 8 weeks were fed standard chow (Oriental Yeast, Tokyo, Japan) and were allowed free access to food and water at room temperature (25 °C) under a 12-h light cycle. The mice were randomly divided into four groups, and treated with the vehicle, linagliptin (Lina), OSI-906, or OSI-906 in combination with linagliptin (OSI-906 + Lina) for 7 days. This study was approved by the Yokohama City University Institutional Animal Care and Use Committee (IACUC) (Permit Number: F-A-14-041, 31 Mar 2014) and was conducted in accordance with the guidelines of the Animal Care Committee of Yokohama City University.

### 4.2. Drugs

OSI-906 (linsitinib) was purchased from MedChem Express (Monmouth Junction, NJ, USA). Eight-week-old mice were orally administered 10 μL/g body weight of water or linagliptin (3 mg/kg/daily, oral gavage) (provided by Boehringer Ingelheim, Ingelheim, Germany) and were also additionally administered 10 μL/g body weight of either the vehicle (30% Solutol HS-15; BASF, Ludwigshafen, Germany) or OSI-906 (45 mg/kg) orally at 30 min after the previous administration once a day for 7 days. Liraglutide was obtained from Novo Nordisk (Bagsværd, Denmark). AML-12 cells were treated with 10 nM linagliptin or 100 nM liraglutide in the presence of 200 nM OSI-906.

### 4.3. Biochemical Parameters

The plasma glucose levels were determined using Glutest Neo Super (Sanwa Chemical Co. Kanagawa, Japan) just before and 4 h after the administration of OSI-906 or the vehicle. Serum insulin levels were determined using an insulin kit (Morinaga Institute of Biological Science, Yokohama, Japan). The glycogen content in the liver was determined using a Determiner-GL-E Kit (Wako Pure Chemical Industries, Osaka, Japan). The serum glutamic pyruvic transaminase (GPT), free fatty acid (FFA), and triglyceride (TG) levels were assayed using enzymatic methods (Wako Pure Chemical Industries, Osaka, Japan).

### 4.4. Histological Analysis

Formalin-fixed, paraffin-embedded liver, and epididymal fat tissue sections were stained with hematoxylin and eosin. Hepatic fibrosis was assessed using Masson–Goldner staining in accordance with the manufacture’s instructions. The liver sections were scored for the severity of steatosis, inflammation, hepatocyte ballooning, and fibrosis in accordance with the scoring method NAFLD activity score (NAS) and fibrosis staging. Briefly, two independent researchers who have enough experiments evaluated the NAS and fibrosis staging as follows: The degree of steatosis (grade 0 ≤ 5%; 1 = 5–33%; grade 2 = 34%–66%; grade 3 ≥ 66%), lobular inflammation (0: no foci, 1 < 2 foci per 200x field, 2: 2 to 4 foci per 200x field, and 3: > 4 foci per 200x field), hepatocyte ballooning (0: None; 1: Rare or few; 2: Many), and fibrosis (0: No fibrosis, 1: Perisinusoidal or periportal fibrosis, 2: Perisinusoidal and portal/perioral fibrosis, 3: Bridging fibrosis, and 4: Cirrhosis)

### 4.5. Real-Time PCR

Total RNA was isolated from the liver using an RNase free DNase and RNeasy Kit (Qiagen, Valencia, CA, USA). cDNA was prepared using High Capacity cDNA Reverse Transcription Kits (Applied Biosystems, Foster City, CA, USA) and was subjected to quantitative PCR using THUNDERBIRD SYBR qPCR Mix (Toyobo Co., Ltd., Osaka, Japan). The data were normalized according to the mRNA expression levels of β-actin and TATA box-binding protein (Tbp). The primer sequences are shown in Supplementary Table S9.

### 4.6. Immunoblotting and Immunopricipitation

For immunoblotting, 40 mg of liver tissues were lysed with T-PER tissue protein extraction reagent (Thermo Fisher Scientific) with protease inhibitor (Nacalai Tesque, Kyoto, Japan) and phosphatase inhibitor (Nacalai Tesque, Kyoto, Japan). After centrifugation, the extracts were subjected to immunoblotting with antibodies to acetyl-lysine (Abcam, ab21623, RRID:AB_446436, 1/1000), perilipin 2 (PROGEN #42, 1/2000), NNMT (Abcam, ab119758, RRID: AB_10902083, 1/600), Sirt1 (Abcam, ab110304, RRID: AB_10864359, 1/1000), Sirt2 (Cell Signaling Technology #12672, RRID: AB_2636961, 1/1000), phospho AMPKα (Thr172) (Cell Signaling Technology #2535, RRID: AB_331250, 1/1000), AMPKα (Cell Signaling Technology #2532, RRID: AB_330331, 1/1000), and glyceraldehyde-3-phosphate dehydrogenase (GAPDH) (Cell Signaling Technology #5174, RRID: AB_10622025, 1/1000). Acetylation of PGC-1α was determined by immunoprecipitation of PGC-1α (Santa-Cruz #518025) with liver lysate followed by immunoblotting for acetylated lysine (Abcam, ab21623). FoxO1 acetylation was also determined by immunoprecipitation of FoxO1 (Cell Signaling, #2880, RRID: AB_2106495) with liver lysate followed by immunoblotting for acetylated lysine (Abcam, ab21623). Briefly, antibodies for PGC-1α and FoxO1 were incubated with packed beads (protein L agarose beads; Santa-Cruz Biotechnology, sc-2336) at 4 °C overnight respectively. To prepare tissue lysates, frozen liver tissue was homogenized on ice in lysis buffer. The lysate was incubated with packed protein L beads at 4 °C overnight to minimize nonspecific binding. Pre-cleaned lysates were incubated with respective antibody-coated beads on a rotating incubator overnight at 4 °C. The precipitated complexes were washed in IP buffer and resuspended in loading buffer and boiled for 5 min before western blot assay was performed. Densitometry was performed using Image J software.

### 4.7. Cell Culture

Mouse hepatocyte AML-12 cells were obtained from ATCC (American Type Culture Collection, Manassas, VA, Cat# CRL-2254, RRID:CVCL_0140) and were cultured in Dulbecco’s modified Eagle’s medium/Ham’s F-12 (GIBCO) supplemented with 10% fetal bovine serum (FBS), a mixture of insulin, transferrin, and selenium (ITS; Collaborative Research), and 0.1 μM dexamethasone at 37 °C in a 5% CO_2_ atmosphere. Mycoplasma contamination was not detected by 16s rRNA-based mycoplasma group-specific PCR. AML-12 cells were used between passages 15 and 16.

### 4.8. Sample Preparation for LC-MS/MS

The liver was homogenized in lysis buffer containing 50 mM NH_4_HCO_3_, 8 M urea, 4% deoxycholic acid, 1% phosphatase inhibitor cocktail 2 (Sigma-Aldrich, St. Louis, MO, USA), 1% phosphatase inhibitor cocktail 3 (Sigma-Aldrich), and protease inhibitor cocktail (Roche, Penzberg, Germany). Lysate was obtained after centrifugation at 15,000 rpm for 10 min at 4 °C and precipitation with 4× volume of cold acetone, then reconstituted with an appropriate volume of lysis buffer. A total of 110 μg of proteins extracted from each liver sample was reduced with dithiothreitol (10 mM final concentration) and alkylated with iodoacetamide (25 mM final concentration). After dilution with 3× volume of 50 mM NH_4_HCO_3_, the proteins were digested with trypsin (protein-to-enzyme ratio of 20:1) (Promega) for 18 h at 37 °C. The protein digests were desalted using C18 StageTips with C18 Empore disks (3 M, St. Paul, MN, USA) following the removal of sodium deoxycholate (SDC) [44,45]. The eluted peptides were used after being completely lyophilized in a vacuum concentrator. For phosphopeptide enrichment, we used Titansphere Phos-TiO beads (GL Sciences, Tokyo, Japan) according to the manufacturer’s protocol. Enriched phosphopeptides were desalted using C18 StageTips and lyophilized in a vacuum concentrator.

### 4.9. Proteomic and Phosphoproteomic Analyses

Total peptides and phosphopeptides were analyzed using LTQ-Orbitrap Elite (Thermo Fisher Scientific Inc. MA, USA) coupled with a Dionex Ultimate 3000 RSLC nano system (Thermo Fisher Scientific) and a QExactive mass spectrometer (Thermo Fisher Scientific Inc.) coupled with a Dionex Ultimate 3000 RSLC nano system, respectively. Label-free quantitation was performed using Progenesis QI for proteomics software (Nonlinear Dynamics, Newcastle, UK). The samples were divided into four groups: Vehicle, OSI-906, linagliptin, and OSI-906 + linagliptin. Then, the samples were subjected to separate multivariate statistical analyses of the proteomic and phosphoproteomic data. MS/MS ion searches to identify proteins were performed using MASCOT software (version 2.5.1, Matrix Science, London, UK) against the UniProtKB database (Mus musculus, 16,678 sequences, http://www.uniprot.org/). For total peptides, protein identification was performed using the following parameters: Enzyme, trypsin; peptide mass tolerance, 5 ppm; fragment mass tolerance, 0.5 Da; maximum missed cleavages, 2; and variable modifications such as protein N-terminal acetylation, carbamidomethylation of cysteine, N-terminal carbamylation, and oxidation of methionine. For phosphopeptides, protein identification was performed using the following parameters: Enzyme, trypsin; peptide mass tolerance, 5 ppm; fragment mass tolerance, 0.05 Da; maximum missed cleavages, 2; and variable modifications such as protein N-terminal acetylation, carbamidomethylation of cysteine, N-terminal carbamylation, oxidation of methionine, phosphorylation of serine/threonine, and phosphorylation of tyrosine. We used a 1% overall false discovery rate as a cutoff value to export our results from the database search. In addition, peptides that yielded a peptide ion score of greater than or equal to 30 were used for relative quantitation. Proteins or phosphopeptides with significant quantitative changes were selected according to calculations performed using Progenesis QI for proteomics and the following parameters: Analysis of variance (ANOVA) *p*-value < 0.05, and fold change > 1.2.

### 4.10. IPA Functional Enrichment Analysis

Genes mapped from significantly upregulated or downregulated peptides and phosphopeptides were used to identify cellular and molecular processes, pathways, and upstream regulators using ingenuity pathway analysis (IPA) software (QIAGEN Redwood City, CA, USA; http://www.qiagen.com/ingenuity). Genes were queried against the ingenuity knowledge database as the reference set. The regulated proteins, phosphoproteins, and their log2-transformed SILAC ratios were uploaded into the IPA software, and the top canonical pathways associated with the uploaded phosphoproteins were listed along with the *p*-values calculated using a right-tailed Fisher exact test. Upstream regulators refer to the upstream proteins that are responsible for causing changes in the phosphorylation and/or total expression levels of the queried genes/proteins in the dataset. Activation z-scores were calculated using IPA’s z-score algorithm to predict the overall activation or inhibition of the identified functional cellular processes/pathways and upstream regulators. A positive z-score (z-score > 0) implies an overall predicted activation of the process/pathway/upstream regulator, whereas a negative z-score (z-score < 0) implies an overall predicted inhibition or downregulation of the pathway/process/upstream regulator. Z-scores of ≥ 2 or ≤ −2 were considered by IPA to predict significant activation or inhibition, respectively. Cellular processes/upstream regulators with no z-scores imply that IPA was unable to generate prediction states for these functionalities.

### 4.11. Statistical Analyses

Statistical analyses were performed using SPSS statics 19 (IBM SPSS, Chicago, IL, USA). Numbers for every experiment are given in the figure legends. All the data were expressed as the means ± SEM. To elucidate differences among groups, ANOVA with an additional Tukey–Kramer post-hoc test, repeated measures ANOVA followed by a Bonferroni multiple comparison test, or a Kruskal–Wallis test were used. Statistical methods used for each data were described in the figure legends. Differences with *p*-values < 0.05 (*) or < 0.01 (**) were considered significant.

## Supplementary Materials

Supplementary materials can be found at https://www.mdpi.com/1422-0067/21/21/7815/s1.

## Abbreviations

DIO	Diet-induced obesity
DPP-4	Dipeptidyl peptidase-4
IGF1R	IGF-1 receptor
IR	Insulin receptor
NAFLD	Non-alcoholic fatty liver disease
