# Supplementary material for: Linagliptin Ameliorates Hepatic Steatosis via Non-Canonical Mechanisms in Mice Treated with a Dual Inhibitor of Insulin Receptor and IGF-1 Receptor
Source: Int J Mol Sci. 2020 Oct 22;21(21):7815. doi: 10.3390/ijms21217815 (PMC7672621; doi:10.3390/ijms21217815)
Supplement: Supplementary file 1 [file ijms-21-07815-s001.zip › Supplemenatry Material.pdf]

**Supplementary Materials**

**Supplementary Figure 1**

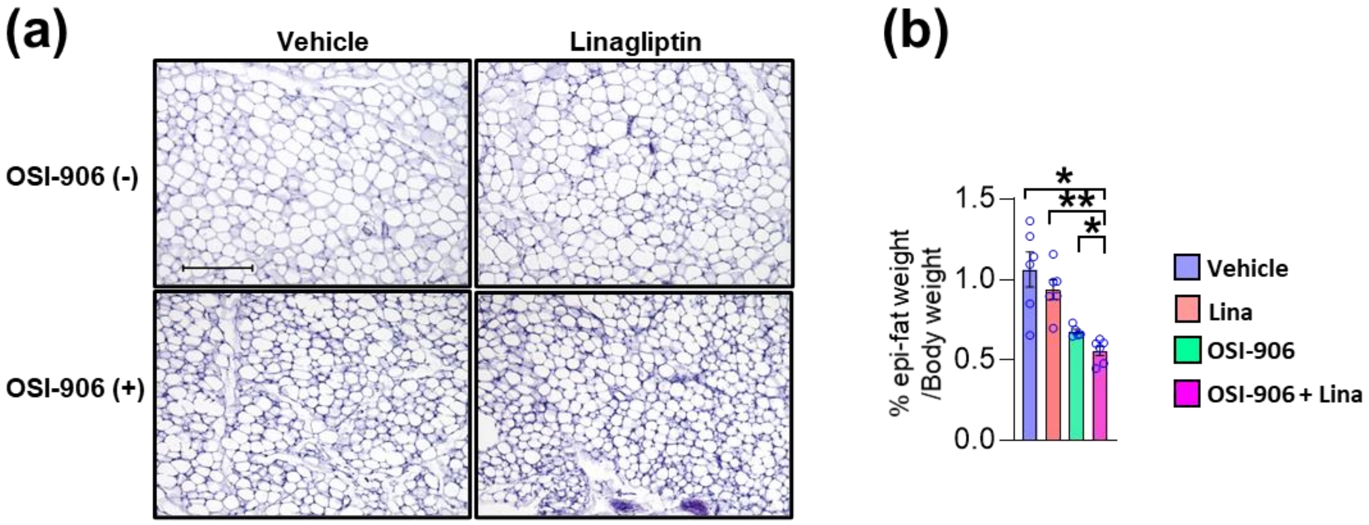


**Supplementary Figure 1.** Linagliptin did not improve lipoatrophy evoked by OSI-906. (**a**) Hematoxylin and eosin-stained sections of epididymal fat on day 7. Scale bar = 200 μm. (**b**) Ratio of epididymal fat weight to body weight on day 7. Data represent the mean ± SEM. *$P < 0.05$, **$P < 0.01$ ($n = 5$-6 per group) by ANOVA with an additional Tukey-Kramer post-hoc test.

Supplementary Figure 2

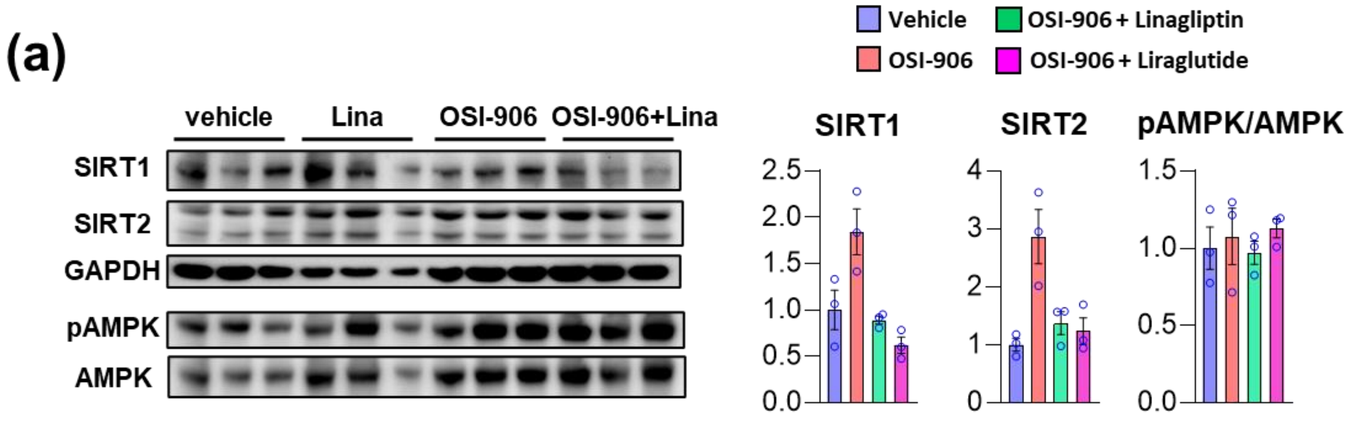


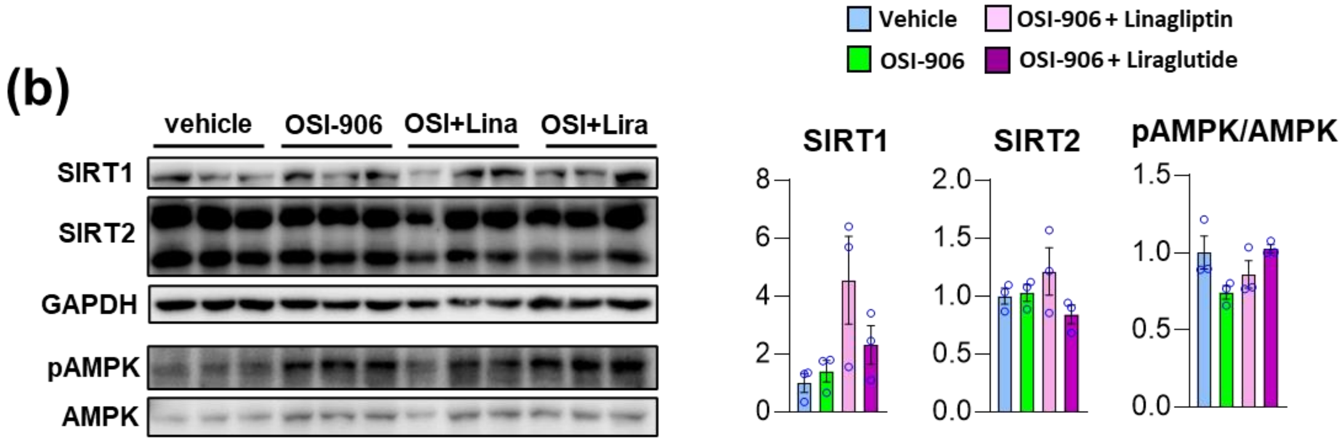


**Supplementary Figure 2.** Sirtuin expression and phosphorylation in AMPK in the liver and AML-12 cells.

(**a**) Immunoblotting for SIRT1, SIRT2, and phosphorylation in AMPK in the liver. Densitometry of each protein was described in right graphs (*n* = 3 per group). (**b**) Immunoblotting for SIRT1, SIRT2, and phosphorylation in AMPK in AML-12 cells treated with 10 nM linagliptin or 100 nM liraglutide in the presence of 200 nM OSI-906 are shown. Cells were serum-starved overnight and then incubated with OSI-906 for 4 hours before the treatment with linagliptin or liraglutide. Then, the cells were incubated for 24 hours with the indicated drugs. Densitometry of each protein was described in right graphs (*n* = 3 per group). Data represent the mean ± SEM.

**Supplementary Figure 3**

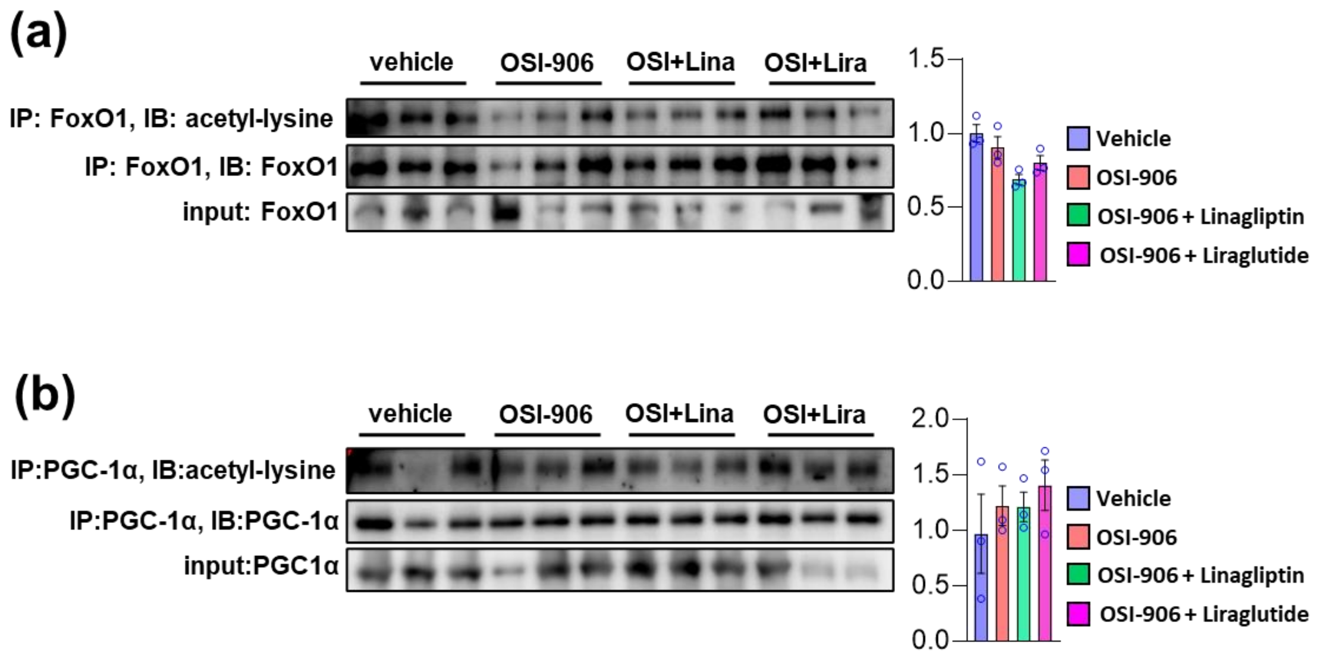


**Supplementary Figure 3.** Sirtuin deacetylase activity on FoxO1 and PGC-1$\alpha$ in the liver.

(**a**) Immunoblotting for acetylated lysine after immunoprecipitation with FoxO1 in the liver. Densitometry of each protein was described in right graphs ($n$ = 3 per group). (**b**) Immunoblotting for acetylated lysine after immunoprecipitation with PGC-1$\alpha$ in the liver. Densitometry of each protein was described in right graphs ($n$ = 3 per group). Data represent the mean ± SEM.

**Supplementary Table 1.** Proteins with hepatic expressions that were significantly altered by OSI-906 administration and restored by treatment with linagliptin

Protein expressions that were upregulated in OSI-906-treated liver and downregulated by linagliptin treatment

| Protein Description | OSI-906 vs. vehicle | | OSI-906 vs. OSI-906 + Lina | |
|---|---|---|---|---|
| | FC | ANOVA (*P*) | FC | ANOVA (*P*) |
| Cytochrome P450 2B10 | 4.51 | 0.0091 | 3.44 | 0.0272 |
| Perilipin-2 | 3.60 | 0.0007 | 2.19 | 0.0073 |
| Aspartate aminotransferase, cytoplasmic | 2.58 | 0.0000 | 1.38 | 0.0265 |
| Phosphoacetylglucosamine mutase | 1.35 | 0.0296 | 1.31 | 0.0387 |
| Bifunctional ATP-dependent dihydroxyacetone kinase/FAD-AMP Lyase (cyclizing) | 1.92 | <0.0001 | 1.28 | 0.0101 |
| Fibronectin | 1.21 | 0.0489 | 1.22 | 0.0389 |

Proteins expressions that were downregulated in OSI-906-treated liver and upregulated by linagliptin treatment

| Protein Description | OSI-906 vs. vehicle | | OSI-906 vs. OSI-906 + Lina | |
|---|---|---|---|---|
| | FC | ANOVA (*P*) | FC | ANOVA (*P*) |
| Major urinary protein 20 | 3.52 | 0.0038 | 2.23 | 0.0440 |
| Lanosterol 14-alpha demethylase | 2.30 | 0.0065 | 1.89 | 0.0147 |
| Isopentenyl-diphosphate Delta-isomerase 1 | 1.67 | 0.0375 | 1.74 | 0.0041 |
| ORM1-like protein 2 | 2.34 | 0.0013 | 1.57 | 0.0106 |
| Protein YIPF5 | 1.69 | 0.0034 | 1.51 | 0.0370 |
| Sterol-4-alpha-carboxylate 3-dehydrogenase, decarboxylating | 1.38 | 0.0396 | 1.50 | 0.0026 |
| Aldose reductase | 1.32 | 0.0467 | 1.50 | 0.0125 |
| Fetuin-B | 1.67 | 0.0014 | 1.46 | 0.0292 |
| Murinoglobulin-2 | 2.18 | 0.0002 | 1.42 | 0.0146 |
| 60S ribosomal protein L6 | 1.56 | 0.0009 | 1.38 | 0.0196 |
| 7-Dehydrocholesterol reductase | 1.44 | 0.0437 | 1.37 | 0.0422 |
| Pyrethroid hydrolase Ces2a | 1.50 | 0.0026 | 1.33 | 0.0137 |
| RNA-binding protein with multiple splicing 2 | 1.74 | 0.0018 | 1.32 | 0.0416 |
| Thymosin beta-4 | 1.47 | 0.0001 | 1.27 | 0.0261 |
| Vimentin | 1.36 | 0.0368 | 1.27 | 0.0370 |
| Carboxylesterase 1D | 1.58 | 0.0011 | 1.23 | 0.0284 |
| UDP-glucuronosyltransferase 2B17 | 1.32 | 0.0141 | 1.22 | 0.0194 |
| 60S ribosomal protein L19 | 1.50 | <0.0001 | 1.22 | 0.0395 |
| Cysteine desulfurase, mitochondrial | 1.25 | 0.0042 | 1.21 | 0.0293 |

FC, fold change

**Supplementary Table 2.** Top upregulated or downregulated proteins in OSI-906-treated liver compared with vehicle-treated liver as revealed by a proteomic analysis

Upregulated in OSI-906-treated liver, compared with vehicle-treated liver

| Protein Description | FC | ANOVA (*P*) |
|---|---|---|
| Steroid 17-alpha-hydroxylase/17,20 lyase | 36.6895 | 0.005451 |
| Cytochrome P450 2B10 | 4.512313 | 0.00908 |
| Nicotinamide N-methyltransferase | 4.330706 | 1.25E-05 |
| Apolipoprotein A-V | 3.775928 | 0.00259 |
| Guanine nucleotide-binding protein G(I)/G(S)/G(O) subunit gamma-1 | 3.627437 | 0.006191 |
| Perilipin-2 | 3.603212 | 0.000742 |
| BRISC complex subunit Abro1 | 2.799342 | 0.004814 |
| Acyl-CoA synthetase short-chain family member 3, mitochondrial | 2.787295 | 0.00223 |
| Aspartate aminotransferase, cytoplasmic | 2.576786 | 1.33E-05 |
| Apolipoprotein A-IV | 2.535404 | 0.004353 |

Downregulated in OSI-906-treated liver, compared with vehicle-treated liver

| Protein Description | FC | ANOVA (*P*) |
|---|---|---|
| Seminal vesicle secretory protein 4 | 22.56498 | 0.030458 |
| Major urinary protein 17 | 4.820739 | 0.047282 |
| Major urinary protein 20 | 3.52338 | 0.003799 |
| Complement component C8 alpha chain | 3.003397 | 0.002165 |
| Developmentally-regulated GTP-binding protein 1 | 2.89517 | 0.047867 |
| Alpha-1-antitrypsin 1-5 | 2.817766 | 0.000491 |
| Cytochrome P450 4A12A | 2.681725 | 0.032885 |
| Bile salt sulfotransferase 2 | 2.597675 | 0.001371 |
| 3 beta-hydroxysteroid dehydrogenase type 5 | 2.5856 | 0.00529 |

FC, fold change

**Supplementary Table 3.** Top upregulated or downregulated proteins in OSI-906 + Lina-treated liver compared with OSI-906-treated liver as revealed by a proteomic analysis

Upregulated in OSI-906 + Lina-treated liver, compared with OSI-906-treated liver

| Protein Description | FC | ANOVA (*P*) |
|---|---|---|
| Major urinary protein 20 | 2.232176 | 0.044048 |
| Lanosterol 14-alpha demethylase | 1.892745 | 0.01474 |
| Isopentenyl-diphosphate Delta-isomerase 1 | 1.743062 | 0.004141 |
| ORM1-like protein 2 | 1.569106 | 0.010644 |
| Protein YIPF5 | 1.509277 | 0.037018 |
| Sterol-4-alpha-carboxylate 3-dehydrogenase, decarboxylating | 1.50342 | 0.002579 |
| Aldose reductase | 1.501844 | 0.012548 |
| Vacuolar protein-sorting-associated protein 25 | 1.499779 | 0.018167 |
| Ubiquinone biosynthesis protein COQ7 homolog | 1.49701 | 0.005744 |
| 60S ribosomal protein L37 | 1.478085 | 0.048925 |
| Fetuin-B | 1.464528 | 0.029177 |
| Transgelin-2 | 1.453406 | 0.026038 |
| Murinoglobulin-2 | 1.42467 | 0.014641 |
| 60S ribosomal protein L6 | 1.3833 | 0.019583 |

Downregulated in OSI-906 + Lina-treated liver, compared with OSI-906-treated liver

| Protein Description | FC | ANOVA (*P*) |
|---|---|---|
| Cytochrome P450 2B10 | 3.442558 | 0.027156 |
| Perilipin-2 | 2.189518 | 0.007271 |
| E3 ubiquitin-protein ligase UBR4 | 1.592003 | 0.03672 |
| Sulfotransferase 1 family member D1 | 1.485781 | 0.041096 |
| Histone H1.5 | 1.423912 | 0.029306 |
| Aspartate aminotransferase, cytoplasmic | 1.382278 | 0.026541 |

FC, fold change

**Supplementary Table 4.** Top upregulated or downregulated phosphopeptides in OSI-906-treated liver compared with vehicle-treated liver as revealed by a phosphoproteomic analysis

Upregulated phosphopeptide in OSI-906-treated liver compared with vehicle-treated liver

| Uniprot ID | Phospho site | Protein Description | FC |
|---|---|---|---|
| Q3UHX2 | S60 | 28 kDa heat- and acid-stable phosphoprotein | Infinity |
| Q8K2K6 | S293 | Arf-GAP domain and FG repeat-containing protein 1 | Infinity |
| Q8K019 | S396 | Bcl-2-associated transcription factor 1 | Infinity |
| O35218 | S419, S420 | Cleavage and polyadenylation specificity factor subunit 2 | Infinity |
| Q5NCI0 | S437 | Up-regulator of cell proliferation | 365.43 |
| P57016 | S62 | Ladinin-1 | 197.61 |
| Q3UPL0 | S526, S530 | Protein transport protein Sec31A | 121.93 |
| Q9CZ44 | S74 | NSFL1 cofactor p47 | 66.80 |
| Q02819 | S85 | Nucleobindin-1 | 64.62 |
| Q9Z131 | S424 | SH3 domain-binding protein 5 | 62.64 |
| Q64511 | S1387 | DNA topoisomerase 2-beta | 54.83 |
| Q63918 | S363 | Serum deprivation-response protein | 47.72 |
| Q9CW07 | S81 | Protein phosphatase 1 regulatory subunit 3G | 38.60 |
| Q8K3J9 | Y386 | G-protein coupled receptor family C group 5 member C | 31.32 |
| Q4FJU9 | S129 | Transmembrane protein 40 | 30.37 |
| E9Q557 | S2620 | Desmoplakin | 25.28 |
| P53996 | S49 | Cellular nucleic acid-binding protein | 20.91 |
| P60904 | S10 | DnaJ homolog subfamily C member 5 | 19.47 |
| P16546 | S1031 | Spectrin alpha chain, non-erythrocytic 1 | 16.66 |
| Q9Z1X4 | S482 | Interleukin enhancer-binding factor 3 | 15.77 |

FC, fold change

**Supplementary Table 5.** Top upregulated or downregulated phosphopeptides in OSI-906-treated liver compared with vehicle-treated liver as revealed by a phosphoproteomic analysis

Downregulated phosphopeptide in OSI-906-treated liver compared with vehicle-treated liver

| Uniprot ID | Phosphosite | Protein Description | FC |
|---|---|---|---|
| Q6PAM0 | S183 | 5'-AMP-activated protein kinase subunit beta-2 | Infinity |
| Q8BP67 | S86 | 60S ribosomal protein L24 | Infinity |
| P28474 | S247 | Alcohol dehydrogenase class-3 | Infinity |
| Q6PEE2 | S72 | CBP80/20-dependent translation initiation factor | Infinity |
| O88735 | T243 | Ensconsin | Infinity |
| Q6P9Q6 | S1159 | FK506-binding protein 15 | Infinity |
| Q6ZQ58 | S824 | La-related protein 1 | Infinity |
| O08638 | S1954 | Myosin-11 | Infinity |
| Q8CES0 | S39 | N-alpha-acetyltransferase 30 | Infinity |
| Q8K3K8 | T210 | Optineurin | Infinity |
| A2AJ88 | S377 | Patatin-like phospholipase domain-containing protein 7 | Infinity |
| Q3U1F9 | S351 | Phosphoprotein associated with glycosphingolipid-enriched microdomains 1 | Infinity |
| P60335 | S190 | Poly(rC)-binding protein 1 | Infinity |
| Q8K1L5 | S82 | Protein phosphatase 1 regulatory subunit 11 | Infinity |
| Q3TSG4 | S385 | RNA demethylase ALKBH5 | Infinity |
| O89032 | S812 | SH3 and PX domain-containing protein 2A | Infinity |
| Q1HFZ0 | S723 | tRNA (cytosine(34)-C(5))-methyltransferase | Infinity |
| Q6P4T2 | S225 | U5 small nuclear ribonucleoprotein 200 kDa helicase | Infinity |
| Q9CQU5 | S216 | ZW10 interactor | Infinity |
| P56212 | S62 | cAMP-regulated phosphoprotein 19 | 784.37 |

FC, fold change

**Supplementary Table 6.** Top upregulated or downregulated phosphopeptides in OSI-906 + Lina-treated liver compared with OSI-906-treated liver as revealed by a phosphoproteomic analysis.

Upregulated phosphopeptide in OSI-906 + Lina-treated liver compared with OSI-906-treated liver

| Uniprot ID | Phosphosite | Protein Description | FC |
|---|---|---|---|
| Q99J36 | S8 | THUMP domain-containing protein 1 | Infinity |
| Q8K3K8 | T210 | Optineurin | Infinity |
| Q8VCB3 | S8 | Glycogen [starch] synthase, liver | Infinity |
| P14869 | S304 | 60S acidic ribosomal protein P0 | 1302.87 |
| P11352 | S201 | Glutathione peroxidase 1 | 255.83 |
| Q8K2C9 | S114 | Very-long-chain (3R)-3-hydroxyacyl-CoA dehydratase 3 | 147.00 |
| O88622 | S311 | Poly(ADP-ribose) glycohydrolase | 104.31 |
| C0HKD8 | T267 | Microfibrillar-associated protein 1 | 42.10 |
| P24788 | S270 | Cyclin-dependent kinase 11B | 37.66 |
| Q1HFZ0 | S723 | tRNA (cytosine(34)-C(5))-methyltransferase | 24.68 |
| Q3UIR3 | S4 | E3 ubiquitin-protein ligase DTX3L | 22.55 |
| Q3UPL0 | S526 | Protein transport protein Sec31A | 15.80 |
| Q8R0W0 | S3222 | Epiplakin | 12.02 |
| Q5XG73 | S184 | Acyl-CoA-binding domain-containing protein 5 | 11.86 |
| P07901 | S194 | Heat shock protein HSP 90-alpha | 10.32 |
| Q91YI0 | S5 | Argininosuccinate lyase | 9.05 |
| Q61586 | S687 | Glycerol-3-phosphate acyltransferase 1, mitochondrial | 7.97 |
| Q9D0F9 | S117 | Phosphoglucomutase-1 | 7.46 |
| Q8K2C9 | S114 | Very-long-chain (3R)-3-hydroxyacyl-CoA dehydratase 3 | 7.31 |
| Q3THG9 | S409 | Alanyl-tRNA editing protein Aarsd1 | 6.72 |

FC, fold change

**Supplementary Table 7.** Top upregulated or downregulated phosphopeptides in OSI-906 + Lina-treated liver compared with OSI-906-treated liver as revealed by a phosphoproteomic analysis.

Downregulated phosphopeptide in OSI-906 + Lina-treated liver compared with OSI-906-treated liver

| Uniprot ID | Phosphosite | Protein Description | FC |
|---|---|---|---|
| Q7TN98 | S99 | Cytoplasmic polyadenylation element-binding protein 4 | Infinity |
| Q91YR1 | S143 | Twinfilin-1 | Infinity |
| Q3TLH4 | S189, S191 | Protein PRRC2C | Infinity |
| Q8BWW4 | S578 | La-related protein 4 | Infinity |
| P59242 | S134 | Cingulin | Infinity |
| P29699 | S138 | Alpha-2-HS-glycoprotein | Infinity |
| D3Z6Q9 | S443 | Bridging integrator 2 | Infinity |
| P51954 | S1071 | Serine/threonine-protein kinase Nek1 | Infinity |
| P11679 | S336 | Keratin, type II cytoskeletal 8 | 1286.64 |
| Q8CI51 | S228 | PDZ and LIM domain protein 5 | 68.39 |
| Q8K2C9 | S114 | Very-long-chain (3R)-3-hydroxyacyl-CoA dehydratase 3 | 64.81 |
| Q9JLV1 | S360 | BAG family molecular chaperone regulator 3 | 22.55 |
| P51859 | T200 | Hepatoma-derived growth factor | 13.39 |
| P47955 | S101, S104 | 60S acidic ribosomal protein P1 | 11.59 |
| Q99K28 | S339 | ADP-ribosylation factor GTPase-activating protein 2 | 7.58 |
| P70302 | S519 | Stromal interaction molecule 1 | 7.40 |
| P27546 | S901 | Microtubule-associated protein 4 | 5.84 |
| Q8BI08 | S2 | Protein MAL2 | 4.72 |
| Q9JIX8 | S825 | Apoptotic chromatin condensation inducer in the nucleus | 2.56 |
| Q6ZQ58 | T626 | La-related protein 1 | 2.28 |

FC, fold change

**Supplementary Table 8.** Top upstream regulators as revealed by a proteomic analysis

OSI-906 vs. Vehicle

| Top Upstream Regulators | $-LOG_{10}$ (*P*-value) | Predicted Activation |
|---|---|---|
| PPARA | 9.436518915 | Inhibited |
| bezafibrate | 6.28567024 | Inhibited |
| XBP1 | 6.262012674 | Activated |
| pirinixic acid | 5.747146969 | Inhibited |
| ciprofibrate | 5.329754147 | Inhibited |

OSI-906 + Lina vs. OSI-906

| Top Upstream Regulators | $-LOG_{10}$ (*P*-value) | Predicted Activation |
|---|---|---|
| SREBF2 | 9.436518915 | Inhibited |
| SH3TC2 | 6.28567024 | |
| lysophosphatidylcholine | 6.262012674 | Inhibited |
| AGN194204 | 5.747146969 | |
| NFE2L2 | 5.329754147 | |

**Supplementary Table 9.** Primer sequence for real-time PCR

| Gene Name | Forward | Reverse |
|---|---|---|
| Ccl2 | 5′-TTAAAAACCTGGATCGGAACCAA-3′ | 5′-GCATTAGCTTCAGATTTACGGGT-3′ |
| Cd36 | 5′-CCTTAAAGGAATCCCCGTGT-3′ | 5′-TGCATTTGCCAATGTCTAGC-3′ |
| Cyp2b10 | 5′-CAATGTTTAGTGGAGGAACTGCG-3′ | 5′-CACTGGAAGAGGAACGTGGG-3′ |
| Fas | 5′-CCTGGATAGCATTCCGAACCT-3′ | 5′-AGCACATCTCGAAGGCTACACA-3′ |
| Gck | 5′-GCGGAGATGCTCTTTGAC-3′ | 5′-GTCCCACGATGTTGTTCC-3′ |
| G6pase | 5′-TCTGTCCCGGATCTACCTTG-3′ | 5′-GAAAGTTTCAGCCACAGCAA-3′ |
| ICAM-1 | 5′-AGATCACATTCACGGTGCTG-3′ | 5′-CTTCAGAGGAGGAAACAGG-3′ |
| Il-6 | 5′-TAGTCCTTCCTACCCCAATTTCC-3′ | 5′-TTGGTCCTTAGCCACTCCTTC-3′ |
| iNOS | 5′-CCCTTCCGAAGTTTCTGGCAGCAGC-3′ | 5′-GGCTGTCAGAGAGCCTCGTGGCTTTGG-3′ |
| NNMT | 5′-AGGAACCAGGAGCCTTTGACT-3′ | 5′-CCTGAGGGCAGTGCGATAGG-3′ |
| PAI-1 | 5′-GACACCCTCAGCATGTTCATC-3′ | 5′-AGGGTTGCACTAAACATGTCAG-3′ |
| Pepck | 5′-CTGCATAACGGTCTGGACTTC-3′ | 5′-CAGCAACTGCCCGTACTCC-3′ |
| Perilipin 2 | 5′-GTGGAAAGGACCAAGTCTGTG-3′ | 5′-GACTCCAGCCGTTCATAGTTG-3′ |
| Pgc1a | 5′-AACCACACCCACAGGATCAGA-3′ | 5′-TCTTCGCTTTATTGCTCCATGA-3′ |
| Ppara | 5′-ACGATGCTGTCCTCCTTGATG-3′ | 5′-GTGTGATAAAGCCATTGCCGT-3′ |
| Scd1 | 5′-CCGGAGACCCCTTAGATCGA-3′ | 5′-TAGCCTGTAAAAGATTTCTGCAAACC-3′ |
| Socs3 | 5′-GGGTGGCAAAGAAAAGGAG-3′ | 5′-GTTGAGCGTCAAGACCCAGT-3′ |
| Srebp1c | 5′-GGCACTAAGTGCCCTCAACCT-3′ | 5′-GCCACATAGATCTCTGCCAGTGT-3′ |
| Tbp | 5′-GGGGAGCTGTGATGTGAAGT-3′ | 5′-CCAGGAAATAATTCTGGCTCA-3′ |
| Tnf | 5′-CCCTCACACTCAGATCATCTTCT-3′ | 5′-GCTACGACGTGGGCTACAG-3′ |
