# Supplementary material for: Linagliptin Ameliorates Hepatic Steatosis via Non-Canonical Mechanisms in Mice Treated with a Dual Inhibitor of Insulin Receptor and IGF-1 Receptor
Source: Int J Mol Sci. 2020 Oct 22;21(21):7815. doi: 10.3390/ijms21217815 (PMC7672621; doi:10.3390/ijms21217815)

Uncropped images of immunoblots in figures.

The black boxes indicate the cropped band of each immunoblot presented in the corresponding figures.

Fig. 5a

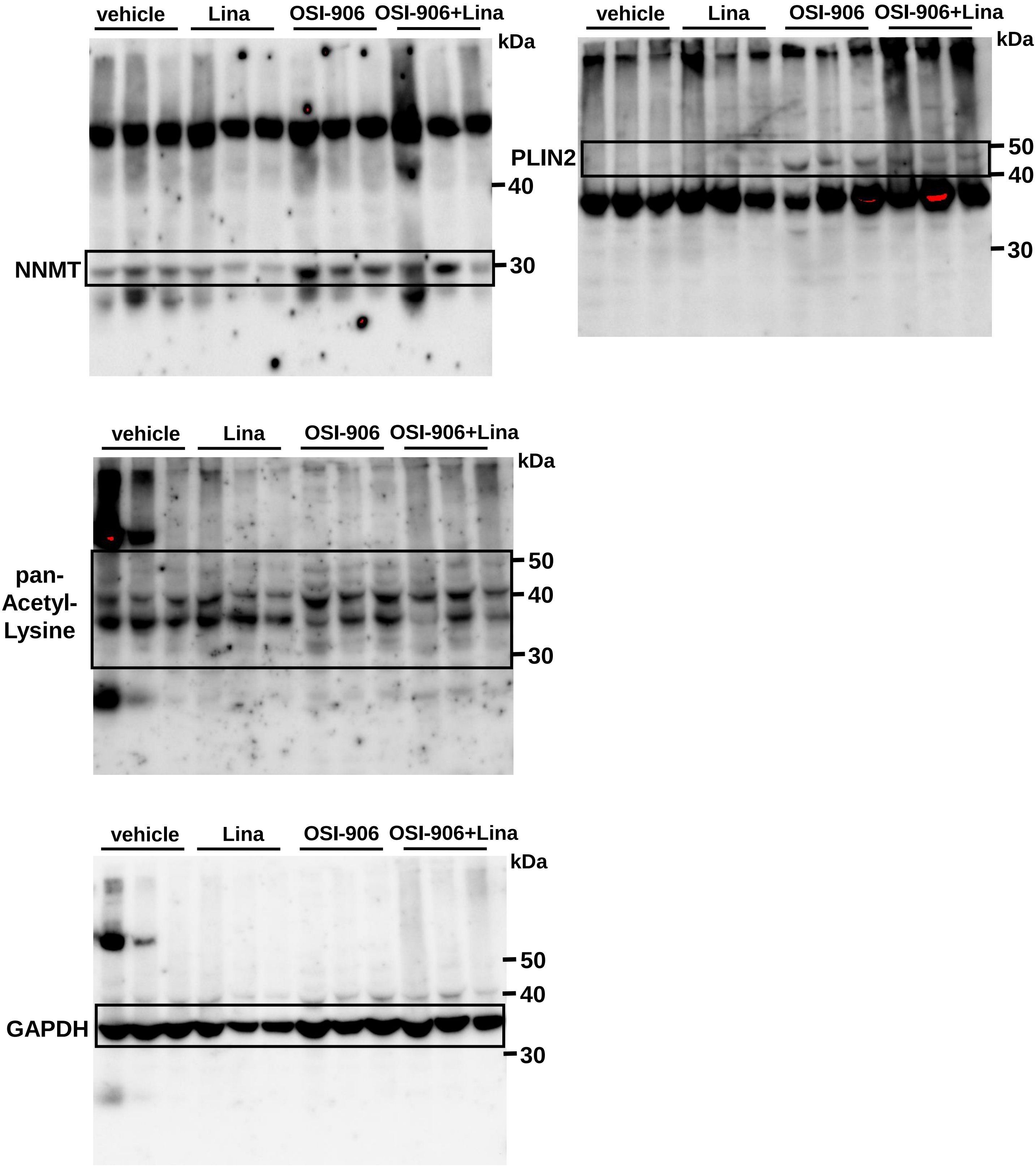

Supplementary Fig. 2a

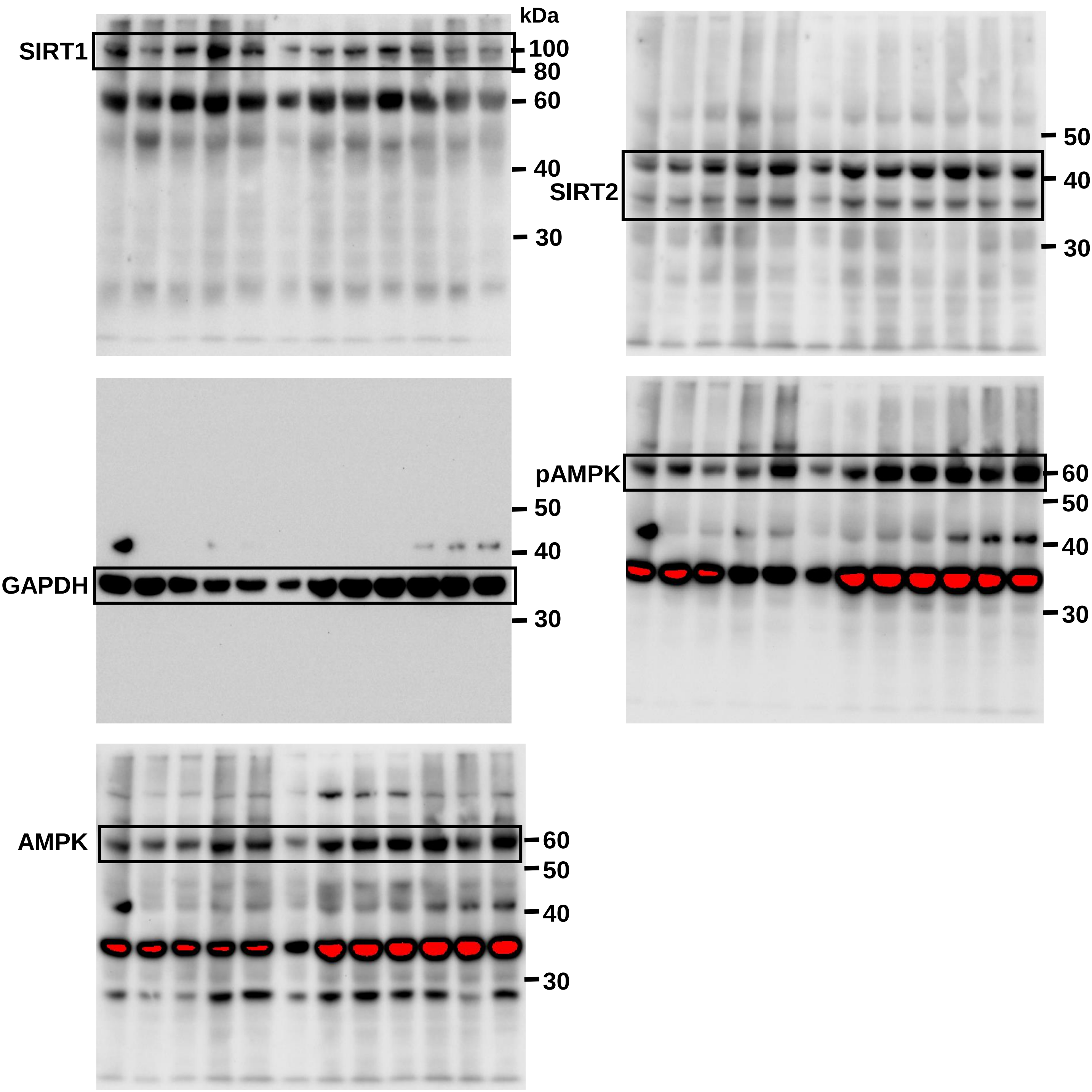

Supplementary Fig. 2b

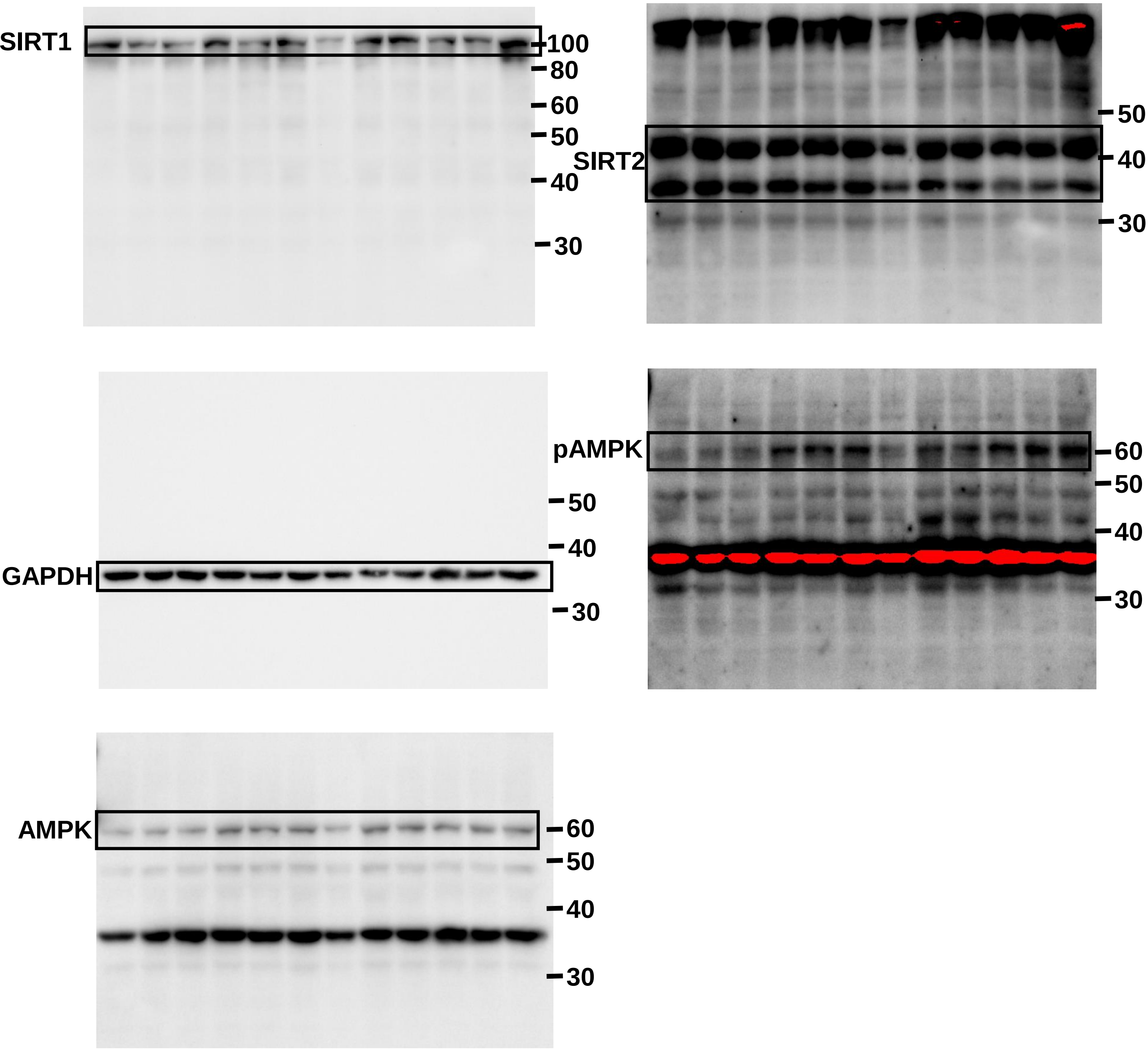

Supplementary Fig. 3

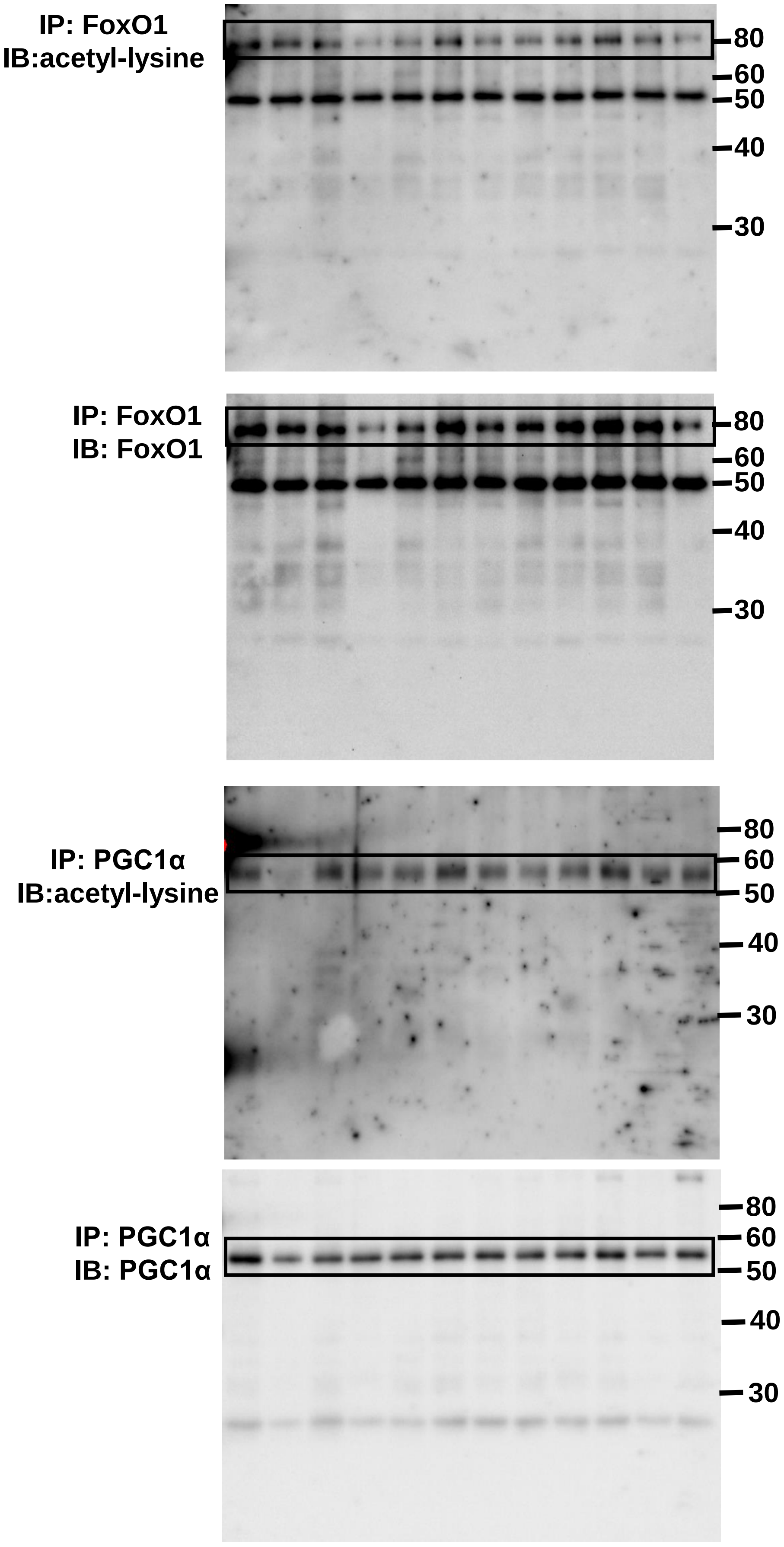

Supplement: Supplementary file 1 [file ijms-21-07815-s001.zip › Uncropped gel.pdf]
